# Supplementary material for: Evaluating access to health and care services during lockdown by the COVID-19 survey in five UK national longitudinal studies
Source: BMJ Open. 2021 Mar 18;11(3):e045813. doi: 10.1136/bmjopen-2020-045813 (PMC7978270; doi:10.1136/bmjopen-2020-045813)
Supplement: Supplementary data [file bmjopen-2020-045813supp001.pdf]

SUPPLEMENTARY MATERIAL FOR:

Evaluating access to health and care services during lockdown by the COVID-19 survey in five UK national longitudinal studies

Constantin-Cristian Topriceanu[1,2], Andy Wong[2], James C Moon[3,4], Alun D Hughes[1,2], David Bann[5], Nish Chaturvedi[1,2], Praveetha Patalay[2,5], Gabriella Conti[6], Gabriella Captur[1,2,7]

Author Affiliations:

1. UCL Medical School, 74 Huntley St, Bloomsbury, London WC1E 6DE
2. UCL MRC Unit for Lifelong Health and Ageing, University College London, Fitzrovia, London WC1E 7HB
3. UCL Institute of Cardiovascular Science, University College London, Gower Street, London WC1E 6BT, UK
4. Cardiac MRI Unit, Barts Heart Centre, West Smithfield, London, EC1A 7BE
5. Centre for Longitudinal Studies, Department of Social Science, University College London, London WC1H 0AL, UK
6. Department of Economics, University College London, London WC1 H0AX, UK
7. The Royal Free Hospital, Centre for Inherited Heart Muscle Conditions, Cardiology Department, Pond Street, Hampstead, London NW3 2QG, UK

Supplementary Table S1. Data missingness per outcome.

**Outcome**	**Cohort study birth year**	**Sex^**	**Ethnicity***	**Socio-economic position†**	**Chronic Illness§**
Cancelled surgery, medical procedures or other medical appointments during lockdown	**1946** (*n*=1170)	0	N/A	64	17
	**1958** (*n*=5073)	0	N/A	761	58
	**1970** (*n*=4099)	0	N/A	578	62
	**1989-1990** (*n*=1849)	0	2	96	49
	**2000-2002** (*n*=2605)	0	19	407	118
Number of care hours during lockdown	**1946** (n=1170)	0	N/A	64	17
	**1958** (n=4884)	0	N/A	731	53
	**1970** (n=3972)	0	N/A	554	60
	**1989-1990** (n=1787)	0	2	98	47
	**2000-2002** (n=2605)	0	19	407	118

n represents the number of individuals for which we had the corresponding outcome, the combined weight and the receipt of a shielding letter.
^ Sex missing value per outcome.
* Ethnicity missing values per outcome.
† Socio-economic position missing values per outcome.
§ Chronic illness missing values per outcome.

1946, refers to National Study of Health and Development (NSHD); 1958, refers to National Child Development Study (NCDS); 1970, refers to British Cohort Study (BCS70); 1989-1990, refers to Next Steps (NS); 2000-2002, refers to Millennium Cohort Study (MCS).

Supplementary Table S2. Chronic Disease breakdown.

Disease	1946	1958	1970	1989-1990	2000-2002
**Cancer**	65	147	45	4	2
**Cystic fibrosis**	0	0	0	0	1
**Asthma**	85	521	498	248	351
**COPD**	45	126	30	1	1
**Wheezy bronchitis**	13	46	21	3	3
**Diabetes**	104	404	156	21	19
**Recurrent backache/prolapsed disc/sciatica/other back problems**	217	838	587	138	104
**Hearing difficulties**	265	606	222	41	46
**Hypertension**	375	1261	443	28	12
**Heart disease**	100	236	43	2	11
**Mental health disease**	59	475	480	286	408
**Obesity**	85	542	439	154	49
**Chronic obstructive airways disease (other than COPD)**	9	23	7	1	1
**Infection**	12	53	30	14	20
**Immunodeficiency (including HIV)**	7	32	28	12	7
**Neurological disease**	24	51	51	12	12

Results are present as counts.

COPD, chronic obstructive pulmonary disease, HIV, human immunodeficiency virus. Other abbreviations as in ***Supplementary Table S1****.*

Supplementary Table S3. Comparison of participants with multiple co-morbidities vs participants with a single chronic disease in terms of the outcomes.

Cohort study birth year	Cancelled surgery, medical procedures or other medical appointments during lockdown^	Number of care hours during lockdown*
**1946**	**0·0001**	0·332
**1958**	**<0·0001**	**<0·0001**
**1970**	**<0·0001**	**<0·0001**
**1989-1990**	0·450	**0·0003**
**2000-2002**	**0·017**	0·196

P-values are presented.
^p-values were obtained from Chi squared test.
*p-values were obtained from Mann-Whitney U test.

Abbreviations as in ***Supplementary Table S1****.*

Supplementary Table S4. Associations of sex with cancelled surgery, medical procedures or other medical appointments during lockdown after adjustment for children under 16 in the household.

**Cohort study birth year**	**Sex^**	
	**OR (95% CI)**	***p*-value**
**1946** (*n*=1154)	0·98 (0·76, 1·25)	0·839
**1958** (*n*=4940)	1·18 (1·01, 1·39)	**0·040**
**1970** (*n*=4008)	1·78 (1·45, 2·21)	**<0·0001**
**1989-1990** (*n*=1796)	1·66 (1·20, 2·34)	**0·003**
**2000-2002** (*n*=2510)	2·29 (1·66, 3·25)	**<0·0001**

Sex was coded as 0=male and 1=female.
All analyses used generalized linear models with logit link. Significant *p*-values are highlighted in bold.
^Adjustment was made for non-response weight, shielding letter and children under 16 in the household.
*CI, confidence interval; OR, odds ratio. Other abbreviations as in **Supplementary Table S1**.*

Supplementary Table S5. Associations of sex with cancelled surgery, medical procedures or other medical appointments during lockdown after adjustment for psychological distress.

**Cohort study birth year**	**Sex^**	
	**OR (95% CI)**	***p*-value**
**1946** (*n*=1059)	1·00 (0·77, 1·30)	0·983
**1958** (*n*=4612)	1·12 (0·95, 1·32)	0·191
**1970** (*n*=3481)	1·82 (1·34, 2·30)	**<0·0001**
**1989-1990** (*n*=1697)	1·71 (1·23, 2·44)	**0·002**
**2000-2002** (*n*=2461)	2·18 (1·55, 3·11)	**<0·0001**

Sex was coded as 0=male and 1=female.
All analyses used generalized linear models with logit link. Significant *p*-values are highlighted in bold.
^Adjustment was made for non-response weight, shielding letter and psychological distress.
*Abbreviations as in **Supplementary Table S4.**.*

Supplementary Table S6. Associations of sex with cancelled surgery, medical procedures or other medical appointments during lockdown after adjustment for chronic illness.

**Cohort study birth year**	**Sex^**	
	**OR (95% CI)**	***p*-value**
**1946** (*n*=1121)	1·01 (0·79, 1·30)	0·822
**1958** (*n*=5015)	1·24 (1·06, 1·46)	**0·007**
**1970** (*n*=4037)	1·79 (1·45, 2·21)	**<0·0001**
**1989-1990** (*n*=1800)	1·64 (1·19, 2·30)	**0·003**
**2000-2002** (*n*=2487)	2·08 (1·51, 2·93)	**<0·0001**

Sex was coded as 0=male and 1=female.
All analyses used generalized linear models with logit link. Significant *p*-values are highlighted in bold.
^Adjustment was made for non-response weight, shielding letter and chronic illness.
*Abbreviations as in **Supplementary Table S4.***

Supplementary Table S7. **Meta-regression for the effect of age on cancelled surgery, medical procedures or other medical appointments and number of care hours needed during lockdown.**

**Analysis**	**Outcome**	**n**	***p*-value^**
Cancelled appointments	Sex	14796	0·196
	Chronic Illness	12584	0·928
Number of care hours	Ethnicity*	4371	N/A
	Chronic Illness	12684	0·352

^p-values for the test of moderators for the restricted cubic spline model are provided.
*Meta-regression was not feasible as only 2 studies recorded ethnicity.
N/A, not available.

Supplementary Table S8. Associations of ethnicity with the number of care hours during lockdown after adjustment for keyworker status.

**Cohort study birth year**	**Ethnicity^**	
	**OR (95% CI)**	***p*-value**
**1989-1990** (*n*=1785)	0·45 (0·28, 0·75)	**0·002**
**2000-2002** (*n*=2586)	0·76 (0·40, 1·63)	0·453

Ethnicity was coded as 0=non-White and 1=White.
All analyses used generalized linear models with logit link. Significant *p*-values are highlighted in bold.
^Adjustment was made for non-response weight, shielding letter, sex and keyworker status.
Abbreviations as in ***Supplementary Table S4.***

Supplementary Table S9. Associations of ethnicity with the number of care hours during lockdown after adjustment for COVID-19 infection.

**Cohort study birth year**	**Ethnicity^**	
	**OR (95% CI)**	***p*-value**
**1989-1990** (*n*=1785)	0·45 (0·28, 0·74)	**0·001**
**2000-2002** (*n*=2586)	0·76 (0·39, 1·60)	0·429

Ethnicity was coded as 0=non-White and 1=White.
All analyses used generalized linear models with logit link. Significant *p*-values are highlighted in bold.
^Adjustment was made for non-response weight, shielding letter, sex and keyworker status.
Abbreviations as in ***Supplementary Table S4.***

Supplementary Table S10. Associations of sex and with cancelled surgery, medical procedures or other medical appointments during lockdown after multiple imputation.

Cohort study birth year	Sex^	
	**OR (95% CI)**	***p*-value**
**1958**	1·19 (1·08, 1·30)	**<0·0001**
**1970**	1·83 (1·61, 2·07)	**<0·0001**
**1989-1990**	1·50 (1·23, 1·83)	**0·002**
**2000-2002**	1·75 (1·32, 2·33)	**0·003**

Sex was coded as 0=male and 1=female.
All analyses used generalized linear models with logit link. Significant *p*-values are highlighted in bold.
^ Adjustment was made for shielding letter.
Abbreviations as in ***Supplementary Table S4.***

Supplementary Table S11. **Association of sex, ethnicity, socio-economic position and the presence of chronic illness with number of care hours during lockdown after further adjustment for hours of care before lockdown.**

Cohort study birth year	Sex^			Ethnicity*			Socio-economic position†			Chronic Illness§		
	**OR (95% CI)**	***p*-value**	**Brant test *p*-value**	**OR (95% CI)**	***p*-value**	**Brant test *p*-value**	**OR (95% CI)**	***p*-value**	**Brant test *p*-value**	**OR (95% CI)**	***p*-value**	**Brant test *p*-value**
**1946** (*n*=1170)	1·25 (0·76, 2·07)	0·377	**<0·0001**	N/A	N/A	N/A	0·85 (0·35, 2·06)	0·718	0·148	1·93 (1·02, 4·00)	**0·048**	0·260
**1958** (*n*=4860)	1·42 (1·02, 1·99)	**0·038**	**0·004**	N/A	N/A	N/A	1·22 (0·66, 2·26)	0·523	**0·046**	1·59 (1·05, 2·45)	**0·031**	**0·015**
**1970** (*n*=3960)	0·91 (0·61, 1·37)	0·654	**<0·0001**	N/A	N/A	N/A	1·37 (0·67, 2·84)	0·389	**<0·0001**	1·75 (1·10, 2·81)	**0·020**	**<0·0001**
**1989-1990** (*n*=1782)	0·60 (0·33, 1·10)	0·097	0·153	0·55 (0·29, 1·11)	0·086	**<0·0001**	1·34 (0·48, 3·83)	0·457	**<0·0001**	1·61 (0·87, 2·98)	0·128	**<0·0001**
**2000-2002** (*n*=2478)	0·95 (0·51, 1·81)	0·868	0·967	0·70 (0·32, 1·76)	0·404	1·00	2·18 (0·76, 6·42)	0·148	0·998	1·21 (0·62, 2·28)	0·562	0·938

Sex was coded as 0=male and 1=female and ethnicity as 0=non-White and 1=White· Socio-economic was coded using childhood social class from 1=managerial to 6=unskilled, but ridit scores were used. Chronic illness was coded as 0=absent and 1=present.
All analyses used generalized linear models with ordinal logit link. Significant *p*-values are highlighted in bold.
^ Adjustment was made for survey non-response weight, previous care hours and shielding letter.
* Adjustment was made for survey non-response weight, previous care hours, shielding letter and sex.
† Adjustment was made for survey non-response weight, previous care hours, shielding letter, sex and ethnicity.
§ Adjustment was made for survey non-response weight, previous care hours shielding letter, sex, ethnicity and SEP.
N/A, not available. Other abbreviations as in ***Supplementary Table S4.***

Supplementary Table S12. **Association of sex, ethnicity, socio-economic position and the presence of chronic illness with number of care hours during lockdown using multinomial logistic regression.**

	**Sex^**			**Ethnicity***			**Socio-economic position[†]**			**Chronic Illness[§]**		
**Cohort study birth year**	**Never[∅]**	**Low RRR [⸍] (95% CI)**	**High RRR [⸌] (95% CI)**	**Never**	**Low RRR (95% CI)**	**High RRR (95% CI)**	**Never**	**Low RRR (95% CI)**	**High RRR (95% CI)**	**Never**	**Low RRR (95% CI)**	**High RRR (95% CI)**
**1946** (*n*=1170)	ref	1·30 (0·79, 2·17)	0·82 (0·38, 1·81)	N/A	N/A	N/A	ref	0·93 (0·39, 2·19)	2·18 (0·55, 8·70)	ref	1·90 (0·97, 3·72)	3·15 (0·93, 10·28)
**1958** (*n*=4884)	ref	1·34 (0·67, 1·50)	1·01 (0·94, 1·92)	N/A	N/A	N/A	ref	1·34 (0·94, 1·91)	1·24 (0·62, 2·48)	ref	**2·27 (1·46, 3·53)**	**2·08 (1·27, 3·40)**
**1970** (*n*=3972)	ref	1·02 (0·66, 1·60)	1·27 (0·72, 2·23)	N/A	N/A	N/A	ref	0·74 (0·35, 1·57)	0·69 (0·27, 1·77)	ref	**2·07 (1·28, 3·36)**	**4·39 (2·18, 8·84)**
**1989-1990** (*n*=1787)	ref	0·49 (0·27, 0·89)	1·11 (0·49, 2·51)	ref	**0·73 (0·20, 0·70)**	0·37 (0·27, 1·99)	ref	0·64 (0·24, 1·76)	1·41 (0·36, 5·43)	ref	1·53 (0·84, 2·78)	1·83 (0·83, 4·05)
**2000-2002** (*n*=2605)	ref	0·50 (0·44, 1·84)	0·90 (0·01, 3·53)	ref	0·92 (0·01, 1·6)	0·19 (0·36, 2·36)	ref	1·98 (0·19, 11·16)	4·62 (0·66, 5·97)	ref	1·08 (0·55, 2·12)	**9·24 (1·03, 83·25)**

Sex was coded as 0=male and 1=female and ethnicity as 0=non-White and 1=White· Socio-economic was coded using childhood social class from 1=managerial to 6=unskilled, but ridit scores were used. Chronic illness was coded as 0=absent and 1=present.
All analyses used multinomial logistic regression.
Statistically significant findings (95% CI exclude the null value) are highlighted in bold.
^ Adjustment was made for survey non-response weight and shielding letter.
* Adjustment was made for survey non-response weight, shielding letter and sex.
[†] Adjustment was made for survey non-response weight, shielding letter, sex and ethnicity.
[§] Adjustment was made for survey non-response weight shielding letter, sex, ethnicity and SEP.
[∅] Defined as 0 hours of care needed post-COVID-19.
[⸍] Defined as 1-9 hours of care needed post-COVID-19.
[⸌] Defined as 10+ hours of care needed post-COVID-19.
*ref, reference; RRR, relative risk ratio. Other abbreviations as in **Supplementary Table S4.***

Supplementary Table S13. Association of socio-economic position surrogates with cancelled surgery, medical procedures or other medical appointments during lockdown.

Cohort study birth year	SEP as highest educational attainment ridit score^			SEP as pre-COVID financial difficulties ridit score^		
	n	**OR (95% CI)**	***p*-value**	***n***	**OR (95% CI)**	***p*-value**
**1946**	1089	1·43 (0·92, 2·21)	0·111	1145	1·28 (0·84, 1·97)	0·246
**1958**	4972	1·09 (0·83, 1·42)	0·559	4878	1·11 (0·85, 1·46)	0·454
**1970**	3850	1·12 (0·80, 1·58)	0·501	3978	1·10 (0·79, 1·54)	0·576
**1989-1990**	1722	1·41 (0·84, 2·37)	0·190	1768	1·49 (0·91, 2·44)	0·119
**2000-2002**	2444	0·84 (0·54, 1·31)	0·477	2413	0·90 (0·58, 1·40)	0·654

All analyses used generalized linear models with logit link.
^ Adjustment was made for survey non-response weight, shielding letter, sex and ethnicity.
*Abbreviations as in **Supplementary Table S4.***

Supplementary Table S14. Association of socio-economic position surrogates with the number of care hours during lockdown.

Cohort study birth year	SEP as highest educational attainment ridit score^			SEP as pre-COVID financial difficulties ridit score^		
	n	**OR (95% CI)**	***p*-value**	***n***	**OR (95% CI)**	***p*-value**
**1946**	1170	1·17 (0·77, 1·79)	0·672	1170	1·18 (0·58, 2·40)	0.657
**1958**	4789	1·12 (0·72, 1·75)	0·609	4836	1·20 (0·77, 1·87)	0·407
**1970**	3747	0·68 (0·39, 1·20)	0·182	3954	0·81 (0·47, 1·39)	0·345
**1989-1990**	1666	0·99 (0·43, 2·25)	0·979	1768	0·92 (0·43, 2·00)	0·841
**2000-2002**	2444	1·53 (0·57, 4·14)	0·408	2420	1·86 (0·73, 4·79)	0·196

All analyses used generalized linear models with ordinal logit link·
^ Adjustment was made for survey non-response weight, shielding letter, sex and ethnicity.
*Abbreviations as in **Supplementary Table S4.***
