# Supplementary material for: Evaluating access to health and care services during lockdown by the COVID-19 survey in five UK national longitudinal studies
Source: BMJ Open. 2021 Mar 18;11(3):e045813. doi: 10.1136/bmjopen-2020-045813 (PMC7978270; doi:10.1136/bmjopen-2020-045813)
Supplement: Author's manuscript [file bmjopen-2020-045813.draft_revisions.pdf]

BMJ Open

BMJ Open is committed to open peer review. As part of this commitment we make the peer review history of every article we publish publicly available.

When an article is published we post the peer reviewers' comments and the authors' responses online. We also post the versions of the paper that were used during peer review. These are the versions that the peer review comments apply to.

The versions of the paper that follow are the versions that were submitted during the peer review process. They are not the versions of record or the final published versions. They should not be cited or distributed as the published version of this manuscript.

BMJ Open is an open access journal and the full, final, typeset and author-corrected version of record of the manuscript is available on our site with no access controls, subscription charges or pay-per-view fees (http://bmjopen.bmj.com).

If you have any questions on BMJ Open's open peer review process please email info.bmjopen@bmj.com

BMJ Open

Inequality in access to health and care services during lockdown – Findings from the COVID-19 survey in five UK national longitudinal studies

Journal:	*BMJ Open*
Manuscript ID	bmjopen-2020-045813
Article Type:	Original research
Date Submitted by the Author:	13-Oct-2020

Complete List of Authors:	Topriceanu, Constantin-Cristian; University College London,<br>Wong, Andrew; UCL, MRC Unit for Lifelong Ageing<br>Moon, James; Barts Heart Centre, Cardiac Imaging Department<br>Hughes, A; University College London<br>Bann, David; University College London,<br>Chaturvedi, Nishi; University College London,<br>Patalay, Praveetha ; UCL<br>Conti, Gabriella; UCL<br>Captur, Gaby; University College London

Keywords:	EPIDEMIOLOGY, Quality in health care < HEALTH SERVICES ADMINISTRATION & MANAGEMENT, PUBLIC HEALTH

SCHOLARONE™
Manuscripts

I, the Submitting Author has the right to grant and does grant on behalf of all authors of the Work (as defined in the below author licence), an exclusive licence and/or a non-exclusive licence for contributions from authors who are: i) UK Crown employees; ii) where BMJ has agreed a CC-BY licence shall apply, and/or iii) in accordance with the terms applicable for US Federal Government officers or employees acting as part of their official duties; on a worldwide, perpetual, irrevocable, royalty-free basis to BMJ Publishing Group Ltd ("BMJ") its licensees and where the relevant Journal is co-owned by BMJ to the co-owners of the Journal, to publish the Work in this journal and any other BMJ products and to exploit all rights, as set out in our licence.

The Submitting Author accepts and understands that any supply made under these terms is made by BMJ to the Submitting Author unless you are acting as an employee on behalf of your employer or a postgraduate student of an affiliated institution which is paying any applicable article publishing charge ("APC") for Open Access articles. Where the Submitting Author wishes to make the Work available on an Open Access basis (and intends to pay the relevant APC), the terms of reuse of such Open Access shall be governed by a Creative Commons licence – details of these licences and which Creative Commons licence will apply to this Work are set out in our licence referred to above.

Other than as permitted in any relevant BMJ Author's Self Archiving Policies, I confirm this Work has not been accepted for publication elsewhere, is not being considered for publication elsewhere and does not duplicate material already published. I confirm all authors consent to publication of this Work and authorise the granting of this licence.

For peer review only

Inequality in access to health and care services during lockdown – Findings from the COVID-19 survey in five UK national longitudinal studies

Constantin-Cristian Topriceanu[1,2], Andrew Wong[2], James C Moon[3,4], Alun D Hughes[1,2], David Bann[5], Nish Chaturvedi[1,2], Praveetha Patalay[2,5], Gabriella Conti[6], Gaby Captur[1,2,7]

Author Affiliations:

1. UCL Medical School, 74 Huntley St, Bloomsbury, London WC1E 6DE
2. UCL MRC Unit for Lifelong Health and Ageing, University College London, London WC1E 7HB
3. UCL Institute of Cardiovascular Science, University College London, Gower Street, London WC1E 6BT, UK
4. Cardiac MRI Unit, Barts Heart Center, West Smithfield, London, EC1A 7BE
5. Center for Longitudinal Studies, Department of Social Science, University College London, London WC1H 0AL, UK
6. Department of Economics and UCL Social Research Institute, University College London, London WC1 H0AX, UK
7. The Royal Free Hospital, Center for Inherited Heart Muscle Conditions, Cardiology Department, Pond Street, Hampstead, London NW3 2QG, UK

Corresponding author:

Gabriella Captur
Consultant Cardiologist in Inherited Heart Muscle Conditions,
Senior Clinical Lecturer,
MRC Unit for Lifelong Health and Ageing at University College London,
1-19 Torrington Place,
Fitzrovia, London WC1E 7HB, UK
E-mail: gabriella.captur@ucl.ac.uk, Phone No: +44 207460059

For peer review only

ABSTRACT

Objective: Access to health services and adequate care is influenced by sex, ethnicity, socio-economic position (SEP) and burden of co-morbidities. Our study aimed to assess whether the COVID-19 pandemic further deepened these already existing health inequalities.

Design: Cross-sectional study.

Setting: Data was collected from five longitudinal age-homogenous British cohorts (born in 2001, 1990, 1970, 1958 and 1946).

Participants: A web and telephone-based survey was sent to the cohorts. Anybody who responded to the survey was included resulting in 14891 eligible participants.

Main outcomes measured: The survey provided data on cancelled surgical or medical appointments, and the number of care hours received during the UK COVID-19 national lockdown.

Interventions: Using binary or ordered logistic regression, we evaluated whether these outcomes differed by sex, ethnicity, SEP and having a chronic illness. Adjustment was made for study-design, non-response weights, psychological distress, presence of children or adolescents in the household, keyworker status, and whether participants had received a shielding letter. Meta-analyses were performed across the cohorts and meta-regression evaluated the effect of age as a moderator.

Results: Females (OR 1·40, 95% confidence interval [1·27,1·55]) and those with a chronic illness (OR 1·84 [1·65-2·05]) experienced significantly more cancellations during lockdown (all $p<0 \cdot 0001$). Ethnic minorities and those with a chronic illness required a higher number of care hours during the lockdown (both OR ≈2·00, all $p<0 \cdot 002$). Age was not independently associated with either outcome in meta-regression. SEP was not associated with cancellation or care hours.

Conclusions: The UK government's lockdown approach during the COVID-19 pandemic appears to have deepened existing health inequalities, impacting predominantly females, ethnic-minorities and those with chronic illnesses. Public health authorities need to implement urgent policies to ensure equitable access to health and care for all in preparation for a second wave.

Keywords: access to healthcare; health inequalities, SARS-CoV2 pandemic.

For peer review only - http://bmjopen.bmj.com/site/about/guidelines.xhtml

Summary boxes

Section 1: What is already known about this topic?
Access to health services and adequate care has previously been shown to be influenced by sex, ethnicity, socio-economic position and burden of co-morbidities. A trend towards increased health inequalities in healthcare access brought about by the COVID-19 pandemic has been observed in United States.

Section 2: What this study adds?
To our knowledge, this is the first study exploring these effects in the UK. Our results suggest that the pandemic might have widened pre-existing health care inequalities, further depriving already vulnerable and disadvantaged groups of the health and care services which they need.

Article summary

Strengths and limitations of this study

- Strengths of the study are the implicit age homogeneity of participants enabling age-matching within each cohort as participants were exposed to similar life factors before the national lockdown. Combining five cohorts spanning multiple age groups (19, 30, 50, 62 and 74 years old) enabled a better understanding of how the COVID-19 pandemic has affected different generations.
- Limitations include data missingness due to low response rates, particularly in younger cohorts, and the small sample size of older cohorts, particularly NSHD. However, given the longitudinal nature of the cohorts, all the analyses have been adjusted for via sample weights derived from missingness predictors that would not otherwise be possible for cross-sectional studies.
- We binarized the ethnicity variable to enable sufficient sample sizes for comparisons but this precluded more detailed comparisons between the diverse ethnic groups which exist in the UK.

- Older cohorts (NSHD, NCDS and BCS70) consist of only white participants, so we were unable to describe findings for older persons from minority ethnic groups that may have been most adversely affected by lockdown.
- As self-reported measures were used, the number of care hours needed before and during lockdown were subject to reporting biases. In addition, single categorical outcome variables do not have the capacity to measure the impact spectrum generated by the cancelled appointments as well as the loss of care hours.

For peer review only - http://bmjopen.bmj.com/site/about/guidelines.xhtml

INTRODUCTION

On 11 March 2020, the World Health Organization declared the novel severe acute respiratory syndrome-coronavirus-19 (SARS-CoV-2, also known as COVID-19) outbreak a global pandemic. As the United Kingdom (UK) was facing a surge of new cases, the government-imposed lockdown restrictions across England, Scotland and Wales on 23 March 2020 in order to limit the spread of the virus. Although the restrictions were gradually relaxed, the most widely accepted end-date of the lockdown is considered to be the 4th of July 2020 when non-essential businesses such as bars, restaurants opened. Delivery of routine care across the UK National Health Service was hampered by the pandemic crisis and the lockdown.

Access to health services and adequate care has previously been shown to be influenced by sex, ethnicity, socio-economic position (SEP) and burden of co-morbidities[1,2]. However, it is unknown whether access to health and care services during the COVID-19 pandemic differed by these factors, potentially further widening already existing health inequalities[3]. Evidence from previous pandemics suggests this possibility, but data is missing in the context of COVID-19 currently. To answer these questions, a web-based survey was sent to participants in five UK national longitudinal studies, spanning multiple generations; data were collected during the core UK lockdown, between 2nd of May and 1st of June 2020. We investigated the number of participants having a cancelled surgical or medical appointment and the number of care hours received for self or other household members over a week during the lockdown. We analyzed how these outcomes varied by already established factors contributing to health inequalities. The importance of cancellations stems from the potential consequences of healthcare deprivation, while the number of weekly care hours has been shown to predict admission to long-term care facilities especially in the older population[4].

METHODS

Study design

The five UK national longitudinal studies were: National Study of Health and Development Study (NSHD)[5], National Child Development Study (NCDS)[6], 1970 British Cohort Study (BCS70)[7], Next Steps (NS)[8] and Millennium Cohort Study (MCS)[9]. NSHD participants were born in 1946, NCDC in 1958, BCS70 in 1970 and MCS in 2000-2002 and all participants were followed-up from birth (all birth cohorts), while NS is a longitudinal cohort study whose participants, born in 1989-1990, have been followed-up from adolescence. The cohorts have been extensively followed up with periodic assessments which have been described elsewhere. During the COVID-19 pandemic (May 2020), an identical online questionnaire, which measured demographic, behavioral and health variables, was sent to each participant from each cohort. The questionnaire was designed to explore the health, care, social, economic, behavioral and psychological consequences of the COVID-19 pandemic. The questionnaire format was multiple choice, but participants were also allowed free text entry to describe their experience in their own words. Ethical approval was obtained from the relevant committees and from the University College London/Institute of Education research ethics committee (REC1334). All participants gave informed consent before taking part.

For peer review only - http://bmjopen.bmj.com/site/about/guidelines.xhtml

Outcomes

Cancelled surgery, medical procedures or other medical appointments were recoded as a binary variable (yes/1 or no/0). The number of hours of help received for self or other household member in a week during lockdown was recorded in six categories: 0, 1-4, 5-9, 10-19, 20-34 or 35+ hours.

Exposures

Sex was recoded as 0=male and 1=female, while ethnicity was recoded as 0=non-White and 1=White. As NSHD, NCDS and BCS70 consist mostly of White participants, ethnicity data was examined only for the NS and MCS cohorts. Highest educational attainment and financial difficulties prior to COVID-19 were used as a proxy for adult SEP. Highest educational attainment was categorized as: degree/higher, advanced-level exam/diploma, ordinary-level exam/general certificate of secondary education or none. Financial difficulties before lockdown were self-rated using the following options: managing comfortably, all right, getting by and difficult. As many MCS participants were still undertaking education and financially dependent on their families, their parents' highest education and financial difficulties were used. Childhood social class has also been recorded according to the UK Office of Population Censuses and Surveys Registrar General's social class resulting in six categories: professional, managerial and technical, skilled non-manual, skilled manual, partly-skilled and unskilled.

Participants were asked to report whether they had a long-standing illness (yes/no). In addition, the name of the chronic illness was also recorded. The number of hours of help received for self or another household member in a week before the pandemic was recorded as above. Whether the participant had received a shielding letter was also noted (yes/no). The presence of children aged less than 16 years in the household as well as the self-reported presence of psychological distress during lockdown were recorded (yes/no). The presence of psychological distress was defined as a score of four or over in the General Health Questionnaire (GHQ-12[10]). Keyworker status was self-rated based on whether participants' work was classified as critical to the COVID-19 response.

Statistical analysis

Statistical analysis was performed in R (version-3.6.0). Frequency distribution of continuous data were assessed visually using histograms. Categorical variables were expressed as counts and percent for each available category. Within each cohort, childhood SEP, highest educational attainment and financial difficulties were converted into cumulative rank probabilities (ridit scores) to quantify the difference in outcomes comparing the lowest with highest SEP (i.e, the relative indices of inequality)[11]. Models containing all socio-economic variables were assessed for multicollinearity via the variance inflation method. As childhood SEP was multicollinear with the other two, had the least amount of missing data, and as it could impact on adult behaviors and health outcomes independently of adult SEP[12], it was used in subsequent analysis. However, we additionally report the results from the analyses using SEP based on highest educational attainment, and financial difficulties respectively.

For peer review only - http://bmjopen.bmj.com/site/about/guidelines.xhtml

Separate regression models were using sex, ethnicity, SEP and presence of chronic illness as predictors of cancelled appointments or number of care hours needed during lockdown. Generalized linear models with logit link were employed to predict cancelled appointments, while ordinal logistic regression was used to predict the number of care hours needed. The proportional odds assumption for ordinal logistic regression was tested using a Brant test[13]. The analysis was weighted to reduce biases due to missing data. Weights were constructed from logistic regression models predicting the response during the COVID-19 data sweep using demographic, socioeconomic, household and individual predictors of non-response at previous data collection points[14,15]. We also used weights to account for the stratified survey designs of 1946, 1990 and 2000-2002 cohorts[16]. Predictors were included sequentially one at a time. Sex analyses were adjusted for these survey non-response weights and for receipt of a shielding letter. All other analyses were similarly adjusted, but ethnicity analyses were additionally adjusted for sex, SEP analyses additionally adjusted for sex and ethnicity, and chronic illness analyses additionally adjusted for sex, ethnicity and SEP.

Gender differences were further evaluated by adjusting for children less than 16 years in the household and psychological distress during the lockdown. As females are more likely to have a chronic disease, gender differences were also evaluated after adjustment for the presence of a chronic disease[17]. Ethnicity differences were further explored by adjusting for key worker status as ethnic minorities have been reported to be over-represented as key workers in the literature[18].

Cohort-specific analyses were conducted initially. Meta-analyses were then performed across the cohorts, only if there was a significant result in at least one of the cohorts. Heterogeneity was evaluated using Cochran' Q test and I^2 statistic. As smaller samples have more sampling errors in their effect estimate, larger effect size might emerge[19]. Thus, funnel plot asymmetry was evaluated using Egger's test. Meta-regression was conducted with age/cohort as a moderator in order to determine whether it was a source of heterogeneity. As the associations between age and our outcomes are likely to be non-linear based on visual inspection, we performed the meta-regression using restricted cubic splines modelling[20].

We ran sensitivity analyses in which we: (1) simulated a complete case analysis through multiple imputation to verify the reliability of observed sex-related differences as the majority of our respondents were female. Using the predictive mean matching method, we have generated 5 complete data sets[21] and performed a pooled regression. The models were not further adjusted for non-response weights; (2) adjusted the number of care hours during lockdown analyses for the number of care hours before the pandemic; (3) explored possible deviations from the proportional odds assumption via multinomial logistic regression with the number of care hours grouped into Never (0 hours), Low (1-9 hours) and High (10 hours+).

For peer review only - http://bmjopen.bmj.com/site/about/guidelines.xhtml

RESULTS

Overall 15291 participants (45% of the combined cohorts' participants) responded to the COVID-19 survey as follows: 1241 out of 1842 (NSHD), 5205 out of 8943 (NCDS), 4247 out of 10458 (BCS), 1921 out of 9380 (NS) and 2677 out of 9909 (MCS). Being female, with higher educational attainment, higher income and better self-rated health were associated with higher response rates[16].

Any participant who lacked data for at least one outcome variable was removed leaving 14891 participants that were included in the final analysis (characteristics summarized in **Table 1**). Overall, included participants were more likely to be female, over 50 years of age and of higher educational attainment. Older participants were more likely to have a chronic illness, receive a shielding letter, experience a cancelled appointment and require more care hours during lockdown. The chronic illnesses recorded spanned a variety of medical systems. Across all cohorts, the most prevalent conditions were high blood pressure (10·9%), recurrent back problems (9·9%), asthma (8·9%) and depression (8.6%)

Cancelled surgery, medical procedures or medical appointments during lockdown

In all cohorts except NSHD, female sex was associated with higher odds (ORs range 1·20–2·29, all p<0·021) of cancelled surgery, medical procedures or medical appointments (**Table 2**). Adjusting for the presence of children less than 16 years old (**Supplementary Table S1**) and the self-rated presence of psychological distress during lockdown (**Supplementary Table S2**) attenuated the regression coefficients in most cohorts, but sex differences persisted. All the sex differences persisted after adjusting for the presence of a chronic illness, but most coefficients were attenuated (**Supplementary Table S3**). The meta-analysis revealed a pooled OR of 1·40 (95% confidence interval [CI] 1·27, 1·55) in the absence of funnel plot asymmetry (Egger test, p=0·376, **Table 3**). However, there was considerable heterogeneity between the cohorts (I^2=85·78%, p<0·0001). In each of the cohorts and in the meta-analysis, presence of a chronic illness at baseline was associated with higher odds (pooled OR 1·84 [1·65, 2·05]) of experiencing a cancelled event. The meta-analysis revealed no heterogeneity (I^2= 0·00%, p=0·422) and no evidence of Funnel plot asymmetry when using the standard error as the predictor (Egger test p=0·092). Ethnicity and SEP were not associated with cancellations in any of the cohorts. Age was not significant in the meta-regression (**Supplementary Table S4**). A visual representation of the cancelled surgery, medical procedures or medical appointments by sex, ethnicity and the presence of chronic illness across the 5 UK cohorts is presented in **Figure 1**.

Number of care hours for self or another household member during lockdown

In older cohorts, chronic illness was more prevalent and the association with number of care hours needed was stronger (**Table 4**). In the meta-analysis, higher number of care hours was associated with ethnic minorities (OR 0·53 [0·35, 0·79], I^2=34·17%), and with the presence of chronic illness (OR 2·20 [1·72, 2·56], I^2=13·22%, **Table 5**). After adjusting for keyworker status, significant associations persisted (**Supplementary Table S5**). Sex and SEP were not associated with the number of care hours needed during lockdown. There was no evidence that age contributed to the heterogeneity between

For peer review only - http://bmjopen.bmj.com/site/about/guidelines.xhtml

cohorts from the meta-regression (**Supplementary Table S1**). Visual representation of the data is provided in **Figure 2.**

Sensitivity analysis

Associations between sex and cancelled surgery, medical procedures or other medical appointments persisted after multiple imputation (**Supplementary Table S6**). Adjustment for previous number of care hours attenuated the OR, but the associations mostly persisted (**Supplementary Table S7**). Findings were similar in the multinomial logistic regression when looking at the transition from Never (0 hours) to Low (1-9 hours), but more variability was observed at the transition from Low to High (10 hours +, **Supplementary Table S8**). When using highest educational attainment or financial difficulties before COVID-19 instead of childhood SEP, there were still no significant associations with cancellations (**Supplementary Table S9**) or the number of care hours during lockdown (**Supplementary Table S10**).

DISCUSSION

Statement of principal findings

These data from five UK national longitudinal studies, at the height of the UK national COVID-19 lockdown in May 2020, indicate worrying health inequalities in the access to health and care services–worst hit were females and those with a chronic illness or from ethnic minority groups.

Meaning of the study

Even before the COVID-19 lockdown, persons with chronic illnesses were vulnerable[22,23] and required more access to health services, as well as care from family members, friends and care service providers[24]. The pandemic triggered unprecedented changes affecting healthcare (which shifted to prioritize COVID-19 patients) and socio-economic dynamics (caused by restricted movement, changes to work patterns and remuneration and unstable housing). Our results show that persons with chronic illnesses were twice as likely to have cancelled medical appointments potentially depriving them of vital medical care. They were also twice as likely to require increased number of care hours. Only around 50% of the participants had their care hours expectations met which suggests that a significant proportion were deprived of essential care. Results persisted after adjustment for shielding letter and previous care-hours illustrating their deeply rooted associations with the outcomes. Overall, participants with chronic illnesses received a double-hit with potentially long-lasting effects on their health and wellbeing.

We found that females were more likely to experience cancellations in planned surgery, medical procedures or other medical appointments during lockdown. This could be linked to pre-existing sex inequalities where females adopt a more caring role prioritizing other family members' needs over their own [25]. Sex inequalities during lockdown could also have widened on account of the added childcare responsibilities including home-schooling, being predominantly undertaken by women. Adjusting for the presence of children under 16 in the household attenuated the regression coefficients suggesting this was a likely contributory factor.

For peer review only - http://bmjopen.bmj.com/site/about/guidelines.xhtml

Ethnic minorities were twice as likely to require an increased number of care hours compared to white participants in the younger cohorts. It is likely that the unstable socio-economic landscape dominated by loss of income, unstable housing, increased psychological distress and reduced community support brought about by the lockdown restrictions adversely impacted these communities. Another explanation could stem from the fact that ethnic minorities are over-represented as key workers[18]. To meet the care needs of their communities, they could have been subjected to increased working hours, unusual working environments, stricter work-based controls, and greater exposure to COVID-19, exacerbating both physical and psychological stress. However, our data suggests that ethnic minorities were under-represented as keyworkers.
Rather surprisingly, the meta-regression showed that age was not a predictor for cancellations or accentuated care needs, suggesting an age-homogenous effect of the lockdown across the generations. We expected that older generations being more frail and likely to have received a shielding letter than younger persons, would have required more care for activities of daily-living and clinical appointments during lockdown. The fact that younger generations (NS and MCS) experienced a substantial number of clinic cancellations and heightened care needs during lockdown is a potentially worrisome indication that the disruption caused by lockdown may have had far reaching effects on the health and wellbeing of young people in the UK.

Implications for clinicians and policymakers
As pandemics can be characterized by multiple waves, they can last several years[26]. Given the prospect of a second wave in the autumn-winter period, it is vital that public health authorities implement national interventions to bolster health and care access. In addition, the healthcare disruptions that occurred during the first wave, are expected to lead to a surge in late-presenting conditions such as cancer[27] which will further strain the healthcare system. The challenge facing public health authorities is the need to promote access to healthcare for vulnerable groups on the one hand, whilst minimizing infection exposure on the other. Countries without a free healthcare systems where citizens rely on paid insurance such as the United States are in an even more difficult position[28].

Unanswered questions and future research
Remote healthcare known as telehealth, has been brought forward as a potential solution to the problem of health inequalities in the COVID-19 situation. However, telehealth is fraught with similar digital inequalities that will hamper the provision of equitable access[29,30]. To make telehealth egalitarian, factors contributing to digital inequalities need to be addressed. These include technical hardware disparities (lack of technological equipment, slower internet connections), digital literacy and access to technical support.

Strengths and weaknesses of the study
Strengths of the study are the implicit age homogeneity of participants enabling age-matching within each cohort as participants were exposed to similar life factors before the national lockdown. Combining five cohorts spanning multiple age groups (19, 30, 50, 62 and 74 years old) enabled a better understanding of how the COVID-19 pandemic has affected different generations.

For peer review only - http://bmjopen.bmj.com/site/about/guidelines.xhtml

Limitations include data missingness due to low response rates, particularly in younger cohorts, and the small sample size of older cohorts, particularly NSHD. However, given the longitudinal nature of the cohorts, all the analyses have been adjusted for via sample weights derived from missingness predictors[14,15] that would not otherwise be possible for cross-sectional studies. We binarized the ethnicity variable to enable sufficient sample sizes for comparisons but this precluded more detailed comparisons between the diverse ethnic groups which exist in the UK. Older cohorts (NSHD, NCDS and BCS70) consist of only white participants, so we were unable to describe findings for older persons from minority ethnic groups that may have been most adversely affected by lockdown. As self-reported measures were used, the number of care hours needed before and during lockdown were subject to reporting biases. In addition, single categorical outcome variables do not have the capacity to measure the impact spectrum generated by the cancelled appointments as well as the loss of care hours. We were unable to separate pandemic effects from recognized confounders such as seasonal variation in the number of care hours needed, as well as unobserved confounders. The overall prevalence of outcomes differed between cohorts and this can affect the interpretation of ORs, potentially introducing bias between cohort comparisons. Lastly, a limitation of the restricted cubic spline meta-regression is the number of knots per variable as our study included only 5 cohorts[31].

Strength and weakness in relation to other studies

A trend towards increased health inequalities in healthcare access brought about by the COVID-19 pandemic has been observed in United States where reduced access to healthcare has been observed for ethnic minorities[32] and individuals with disabilities[33]. To the best of our knowledge this is the first study showing similar effects in the UK. In addition, we are the first to address this issue by combining multiple cohorts spanning multiple age groups and with such a high sample size.

CONCLUSION

Individuals with a chronic illness were more likely to experience cancelled healthcare appointments and greater care needs during the UK national lockdown generated by the COVID-19 pandemic. Females experienced reduced access to healthcare, while ethnic minorities required extra care hours. Our results suggest that the pandemic might have widened pre-existing health care inequalities, further depriving already vulnerable and disadvantaged groups of the health and care services which they need. Public health measures should be rapidly implemented to better protect and meet the health and care demands of such at risk groups ahead of a COVID-19 second wave.

DATA AVAILABILITY

NSHD data is available from: https://www.nshd.mrc.ac.uk/data. Data from the remaining cohorts is available from the UK Data Archive: https://www.data-archive.ac.uk.

FUNDING

The study was funded by the Economic and Social Research Council under the Center for Longitudinal Studies, Resource Center 2015-20, grant number ES/M001660/1, and by the Medical Research Council, grant MC_UU_00019/1. G.C. is supported by British Heart Foundation (MyoFit46 Special Programme Grant SP/20/2/34841), the National Institute

For peer review only

For peer review only - http://bmjopen.bmj.com/site/about/guidelines.xhtml

for Health Research Rare Diseases Translational Research Collaboration (NIHR RD-TRC) and by the NIHR UCL Hospitals Biomedical Research Center. J.C.M. is directly and indirectly supported by the UCL Hospitals NIHR BRC and Biomedical Research Unit at Barts Hospital respectively. DB is supported by the Economic and Social Research Council (grant number ES/M001660/1) and by The Academy of Medical Sciences / Wellcome Trust ("Springboard Health of the Public in 2040" award: HOP001/1025). AH receives support from the British Heart Foundation, the Economic and Social Research Council (ESRC), the Horizon 2020 Framework Programme of the European Union, the National Institute on Aging, the National Institute for Health Research University College London Hospitals Biomedical Research Center, the UK Medical Research Council and works in a unit that receives support from the UK Medical Research Council. GC thanks for support the European Research Council under the European Union's Horizon 2020 research and innovation programme (grant agreement No. 819752 DEVORHBIOSHIP – ERC-2018-COG).

ROLE OF THE FUNDING SOURCE

None of the funders was involved in the study design, the collection, the analysis, the interpretation of the data, and in the decision to submit the article for publication.

ACKNOWLEDGEMENTS

We are grateful to all the members of our studies for their contribution to this COVID-19 survey and for their ongoing participation in our studies. We thank the Survey, Data, and Administrative teams at the Center for Longitudinal Studies and Unit for Lifelong Health and Ageing, UCL, for enabling the rapid COVID-19 data collection to take place.

DISCLOSURES

The views expressed in this article are those of the authors who declare that they have no conflict of interest.

AUTHOR CONTRIBUTIONS

All authors contributed significantly to the design, implementation, analysis, interpretation and manuscript writing. The corresponding author attests that all listed authors meet the authorship criteria and that no others meeting the criteria have been omitted.

TRANSPARENCY STATEMENT

The authors affirm that the manuscript is an honest, accurate, and transparent account of the study being reported.

COPYRIGHT/LICENSE FOR PUBLICATION

The Corresponding Author has the right to grant on behalf of all authors and does grant on behalf of all authors, a worldwide license to the Publishers and its licensees in perpetuity, in all forms, formats and media (whether known now or created in the future), to i) publish, reproduce, distribute, display and store the Contribution, ii) translate the Contribution into other languages, create adaptations, reprints, include within collections and create summaries, extracts and/or, abstracts of the Contribution,

For peer review only

iii) create any other derivative work(s) based on the Contribution, iv) to exploit all subsidiary rights in the Contribution, v) the inclusion of electronic links from the Contribution to third party material where-ever it may be located; and, vi) licence any third party to do any or all of the above.

PATIENT AND PUBLIC INVOLVEMENT

It was not appropriate or possible to involve patients or the public in the design, or conduct, or reporting, or dissemination plans of our research.

DISSEMINATION DECLARATION

We plan to disseminate the results to study participants.

REFERENCES

1. Bleich SN, Jarlenski MP, Bell CN, LaVeist TA. Health Inequalities: Trends, Progress, and Policy. *Annual review of public health* 2012; **33**(1): 7-40.
2. Kojima G, Liljas A, Iliffe S. Frailty syndrome: implications and challenges for health care policy. *Risk management and healthcare policy* 2019; **12**: 23-30.
3. Mein SA. COVID-19 and Health Disparities: the Reality of "the Great Equalizer". *Journal of general internal medicine : JGIM* 2020; **35**(8): 2439-40.
4. Aspell N, apos, Sullivan M, et al. Predicting admission to long-term care and mortality among community-based, dependent older people in Ireland. *International journal of geriatric psychiatry* 2019; **34**(7): 999-1007.
5. Kuh D, Pierce M, Adams J, et al. Cohort profile: updating the cohort profile for the MRC National Survey of Health and Development: a new clinic-based data collection for ageing research. *Int J Epidemiol , 40 (1) e1-e9 (2011)* 2011.
6. Power C, Elliott J. Cohort profile: 1958 British birth cohort (National Child Development Study). *International journal of epidemiology* 2006; **35**(1): 34-41.
7. Elliott J, Shepherd P. Cohort profile: 1970 British Birth Cohort (BCS70). *International Journal of Epidemiology* 2006; **35**(4): 836-43.
8. Calderwood L, Sanchez C. Next Steps (formerly known as the Longitudinal Study of Young People in England). *Open health data* 2016; **4**(1): e2-e.
9. Connelly R, Platt L. Cohort profile: UK Millennium Cohort Study (mcs). *International Journal of Epidemiology* 2014; **43**(6): 1719-25.
10. Montazeri A, Harirchi AM, Shariati M, Garmaroudi G, Ebadi M, Fateh A. The 12-item General Health Questionnaire (GHQ-12): translation and validation study of the Iranian version. *Health and quality of life outcomes* 2003; **1**(1): 66-.
11. Bann D, Johnson W, Li L, Kuh D, Hardy R. Socioeconomic inequalities in childhood and adolescent body-mass index, weight, and height from 1953 to 2015: an analysis of four longitudinal, observational, British birth cohort studies. *The Lancet Public Health , 3 (4) (2018)* 2018.
12. Elhakeem A, Cooper R, Bann D, Hardy R. Childhood socioeconomic position and adult leisure-time physical activity: a systematic review. *The international journal of behavioral nutrition and physical activity* 2015; **12**(1): 92.
13. Brant R. Assessing Proportionality in the Proportional Odds Model for Ordinal Logistic Regression. *Biometrics* 1990; **46**(4): 1171-8.

For peer review only

For peer review only - http://bmjopen.bmj.com/site/about/guidelines.xhtml

14. Silverwood RJ, Calderwood, L., Sakshaug, J.W., Ploubidis, G.B. A data driven approach to understanding and handling nonresponse in the Next Steps cohort. London: UCL Centre for Longitudinal Studies: CLS Working Paper 2020/5; 2020.
15. Mostafa T, Narayanan, M., Pongiglione, B., Dodgeon, B., Goodman, A., Silverwood, R.J., and Ploubidis, G.B. Improving the plausibility of the missing at random assumption in the 1958 British birth cohort: A pragmatic data driven approach. CLS Working Paper 2020/6: London: UCL Centre for Longitudinal Studies; 2020.
16. Bann D, Villadsen A, Maddock J, et al. Changes in the behavioural determinants of health during the coronavirus (COVID-19) pandemic: gender, socioeconomic and ethnic inequalities in 5 British cohort studies. medRxiv; 2020.
17. Gordon EH, Peel NM, Samanta M, Theou O, Howlett SE, Hubbard RE. Sex differences in frailty: A systematic review and meta-analysis. *Experimental Gerontology* 2017; **89**: 30-40.
18. CDC. Health Equity Considerations and Racial and Ethnic Minority Groups. 2020. https://www.cdc.gov/coronavirus/2019-ncov/community/health-equity/race-ethnicity.html (accessed 21/08/2020 2020).
19. Sterne JAC, Sutton AJ, Ioannidis JPA, et al. Recommendations for examining and interpreting funnel plot asymmetry in meta-analyses of randomised controlled trials. *BMJ* 2011; **343**(jul22 1): d4002-d.
20. Orsini N, Li R, Wolk A, Khudyakov P, Spiegelman D. Meta-Analysis for Linear and Nonlinear Dose-Response Relations: Examples, an Evaluation of Approximations, and Software. *American journal of epidemiology* 2012; **175**(1): 66-73.
21. Pedersen AB, Mikkelsen EM, Cronin-Fenton D, et al. Missing data and multiple imputation in clinical epidemiological research. *Clinical epidemiology* 2017; **9**: 157-66.
22. Grumbach K. Chronic illness, comorbidities, and the need for medical generalism. *Annals of family medicine* 2003; **1**(1): 4-7.
23. Bayliss EA. Descriptions of Barriers to Self-Care by Persons with Comorbid Chronic Diseases. *Annals of family medicine* 2003; **1**(1): 15-21.
24. Valderas JM, Starfield B, Sibbald B, Salisbury C, Roland M. Defining comorbidity: implications for understanding health and health services. *Annals of family medicine* 2009; **7**(4): 357.
25. SolÉ-AurÓ A, Crimmins EM. Who cares? A comparison of informal and formal care provision in Spain, England and the USA. *Ageing and society* 2014; **34**(3): 495-517.
26. He D, Dushoff J, Day T, Ma J, Earn DJD. Inferring the causes of the three waves of the 1918 influenza pandemic in England and Wales. *Proceedings of the Royal Society B: Biological Sciences* 2013; **280**(1766): 20131345.
27. Maringe C, Spicer J, Morris M, et al. The impact of the COVID-19 pandemic on cancer deaths due to delays in diagnosis in England, UK: a national, population-based, modelling study. *The lancet oncology* 2020; **21**(8): 1023-34.
28. Khatana SAM, Groeneveld PW. Health Disparities and the Coronavirus Disease 2019 (COVID-19) Pandemic in the USA. *Journal of general internal medicine : JGIM* 2020; **35**(8): 2431-2.
29. Beaunoyer E, Dupéré S, Guitton MJ. COVID-19 and digital inequalities: Reciprocal impacts and mitigation strategies. *Computers in human behavior* 2020; **111**: 106424.

For peer review only - http://bmjopen.bmj.com/site/about/guidelines.xhtml

30. Crawford A, Serhal E. Digital Health Equity and COVID-19: The Innovation Curve Cannot Reinforce the Social Gradient of Health. *Journal of medical Internet research* 2020; **22**(6): e19361.
31. Shepherd BE, Rebeiro PF. Assessing and interpreting the association between continuous covariates and outcomes in observational studies of HIV using splines. *Journal of acquired immune deficiency syndromes (1999)* 2017; **74**(3): e60-e3.
32. Racial, Economic and Health Inequality and COVID-19 Infection in the United States. *Respiratory Therapeutics Week* 2020: 408.
33. Okonkwo NE, Aguwa UT, Jang M, et al. COVID-19 and the US response: accelerating health inequities. *BMJ evidence-based medicine* 2020: bmjebm-2020-111426.

FIGURE LEGENDS

Figure 1. Bar charts illustrating the percentages of cancelled surgery, medical appointments or other medical procedures by sex, ethnicity and the presence of chronic illness across the 5 UK longitudinal cohorts, ordered by increasing age of the cohort from left to right. Error bars representing the 95% confidence intervals are also presented.
1946 refers to National Study of Health and Development (NSHD); 1958 refers to National Child Development Study (NCDS); 1970 refers to British Cohort Study (BCS70); 1989-1990 refers to Next Steps (NS); 2000-2002 refers to Millennium Cohort Study (MCS).

Figure 2. Bar charts illustrating the percentage of participants requiring support based on the number of care hours needed during the UK COVID-19 national lockdown stratified by sex, ethnicity and the presence of chronic illness across the cohorts.

For peer review only

For peer review only - http://bmjopen.bmj.com/site/about/guidelines.xhtml

Table 1. Characteristics of participants by cohort.

**Participant characteristics**	**Cohort study birth year**				
	**1946**	**1958**	**1970**	**1989-1990**	**2000-2002**
Sample size					
Questionnaire respondents (*n*=15291)	1241	5205	4247	1921	2677
Included participants (*n*=14891)	1154	5119	4131	1876	2609
Age, years	74	62	50	30-31	19-20
Males, %	607 (51·88)	2432 (47·51)	1708 (41·40)	633 (34·09)	770 (29·51)
Non-white ethnicity, %	N/A	N/A	N/A	361 (19·27)	367 (14·17)
Childhood SEP I-III, %	633 (57·18)	1897 (43·60)	1727 (48·08)	1227 (69·36)	1755 (79·70)
Education, %GCSEs-none	728 (65·88)	2732 (54·44)	2032 (52·41)	838 (37·94)	1375 (56·00)
Chronic Illness, %	728 (65·88)	2732 (54·44)	2032 (52·41)	838 (37·94)	1375 (56·00)
Shielding letter, %	112 (9·61)	334 (6·57)	196 (4·77)	56 (3·00)	60 (2·30)
Psychological distress during lockdown, %	112 (10·43)	544 (14·90)	572 (16·33)	466 (27·07)	437 (17·74)
Presence of children <16yrs, %	0 (0·00)	87 (2·13)	1660 (41·10)	462 (25·37)	15 (0·60)
Key workers, %					
White	9 (0.78)	938 (18.32)	1396 (33.79)	491 (32.41)	179 (7.98)
Non-White	N/A	N/A	N/A	92 (25.49)	16 (4.36)
**Outcomes**					
Cancelled appointments, %	376 (32·58)	775 (15·17)	494 (11·97)	234 (12·47)	303 (11·61)
Care hours during lockdown					
0 hours	1073	4651	3825	1724	2552
1-4 hours	61	112	66	47	47
5-9 hours	10	41	36	10	4
10-19 hours	8	42	20	17	3
20-34 hours	5	18	16	4	2
35+ hours	13	54	35	10	1

1946 refers to National Study of Health and Development (NSHD); 1958 refers to National Child Development Study (NCDS); 1970 refers to British Cohort Study (BCS70); 1989-1990 refers to Next Steps (NS); 2000-2002 refers to Millennium Cohort Study (MCS); GCSE, general certificate of secondary education; N/A, not available; SEP, socio-economic position; yrs, years.

For peer review only

Table 2. Association of sex, ethnicity, SEP and the presence of chronic illness with cancelled surgery, medical procedures or other medical appointments during lockdown.

Cohort study birth year	Sex^		Ethnicity*		Socio-economic position[†]		Chronic Illness[§]	
	OR (95% CI)	*p*-value	OR (95% CI)	*p*-value	OR (95% CI)	*p*-value	OR (95% CI)	*p*-value
**1946** (*n*=1170)	0·97 (0·76, 1·25)	0·827	N/A	N/A	1·39 (0·90, 2·16)	0·138	1·74 (1·28, 2·36)	**0·0004**
**1958** (*n*=5073)	1·20 (1·03, 1·40)	**0·021**	N/A	N/A	1·05 (0·78, 1·41)	0·753	2·15 (1·76, 2·62)	**<0·0001**
**1970** (*n*=4099)	1·83 (1·47, 2·26)	**<0·0001**	N/A	N/A	1·05 (0·73, 1·51)	0·786	1·77 (1·42, 2·21)	**<0·0001**
**1989-1990** (*n*=1849)	1·70 (1·23, 2·35)	**0·001**	1·25 (0·86, 2·37)	0·255	1·45 (0·88, 2·41)	0·154	1·59 (1·18, 2·13)	**0·002**
**2000-2002** (*n*=2605)	2·29 (1·65, 3·19)	**<0·0001**	1·03 (0·73, 2·31)	0·885	1·05 (0·66, 1·67)	0·836	1·71 (1·30, 2·25)	**0·0001**

All analyses used generalized linear models with logit link. Significant *p*-values are highlighted in bold.
^ Sex was coded as 0=male and 1=female; adjustment was made for survey non-response weight and shielding letter.
* Ethnicity was coded as 0=non-White and 1=White; adjustment was made for survey non-response weight, shielding letter and sex. All participants in NSHD, NCDS and BCS were White, so ethnicity was not examined.
[†] Socio-economic position was coded using childhood social class from 1=managerial to 6=unskilled, but ridit scores were used; adjustment was made for survey non-response weight, shielding letter, sex and ethnicity.
[§] Chronic illness was coded as 0=absent and 1=present; adjustment was made for survey non-response weight, shielding letter, sex, ethnicity and SEP.
*OR, odds ratio; CI, confidence interval. Other abbreviations as in **Table 1**.*

Table 3. Meta-analysis for the respective association of sex and presence of chronic illness with cancelled surgery, medical procedures or other medical appointments during lockdown.

Predictor	*n*	Study Heterogeneity			OR (95%CI)	*p*-value	Egger-test *p*-value
		I^2	Q	*p*-value			
Sex	14796	85·78%	28·12	**<0·0001**	1·40 (1·27, 1·55)	**<0·0001**	0·376
Chronic illness	12584	0·00%	3·89	0·422	1·84 (1·65, 2·05)	**<0·0001**	0·092

*Abbreviations as in **Table 1**.*

For peer review only - http://bmjopen.bmj.com/site/about/guidelines.xhtml

Table 4. Association of sex, ethnicity, socio-economic position and the presence of chronic illness with number of care hours during lockdown.

Cohort study birth year	Sex^			Ethnicity*			Socio-economic position†			Chronic Illness§		
	OR (95% CI)	*p*-value	Brant test *p*-value	OR (95% CI)	*p*-value	Brant test *p*-value	OR (95% CI)	*p*-value	Brant test *p*-value	OR (95% CI)	*p*-value	Brant test *p*-value
**1946** (n=1170)	1·17 (0·77, 1·79)	0·452	**<0·0001**	N/A	N/A	N/A	1·17 (0·56, 2·45)	0·683	0·329	2·20 (1·22, 3·99)	**0.009**	0·192
**1958** (n=4884)	1·25 (0·70, 1·61)	0·087	0·656	N/A	N/A	N/A	1·29 (0·81, 2·06)	0·282	0·877	2·17 (1·56, 3·04)	**<0·0001**	0·860
**1970** (n=3972)	0·99 (0·72, 1·36)	0·955	**0·010**	N/A	N/A	N/A	0·72 (0·39, 1·30)	0·272	0·026	2·74 (1·84, 4·08)	**<0·0001**	0·244
**1989-1990** (n=1787)	0·69 (0·44, 1·08)	0·102	**<0·0001**	0·44 (0·27, 0·72)	**0·001**	**0·005**	0·87 (0·39, 1·94)	0·727	**0·0003**	1·63 (1·01, 2·65)	**0·047**	**0·010**
**2000-2002** (n=2605)	0·88 (0·49, 1·64)	0·681	0·998	0·76 (0·38, 1·53)	0·432	0·972	2·17 (0·77, 6·25)	0·146	0·998	1·38 (0·75, 2·56)	0·301	0·961

All analyses used generalized linear models with ordinal logit link. Socio-economic was recorded using childhood social class Significant *p*-values are highlighted in bold.
^ Sex was coded as 0=male and 1=female; adjustment was made for survey non-response weight and shielding letter.
* Ethnicity was coded as 0=non-White and 1=White; adjustment was made for survey non-response weight, shielding letter and sex. All participants in NSHD, NCDS and BCS were White, so ethnicity was not examined.
† Socio-economic position was coded using childhood social class from 1=managerial to 6=unskilled, but ridit scores were used; adjustment was made for survey non-response weight, shielding letter, sex and ethnicity.
§ Chronic illness was coded as 0=absent and 1=present; adjustment was made for survey non-response weight, shielding letter, sex, ethnicity and SEP.
Abbreviations as in ***Table 1***.

For peer review only

Table 5. **Meta-analysis for the respective association of sex and presence of chronic illness with number of care hours during lockdown.**

Predictor	*n*	Study Heterogeneity			OR (95%CI)	*p*-value	Egger-test *p*-value
		I^2	Q	*p*-value			
Ethnicity	4371	34·17%	0·218	0·218	0·53 (0·35, 0·79)	**0·002**	N/A^
Chronic illness	12684	13·22%	4·609	0·330	2·10 (1·72, 2·56)	**<0·0001**	0·312

^Egger test was not feasible as only 2 studies recorded ethnicity.
Abbreviations as in ***Table 1***.

For peer review only

For peer review only - http://bmjopen.bmj.com/site/about/guidelines.xhtml

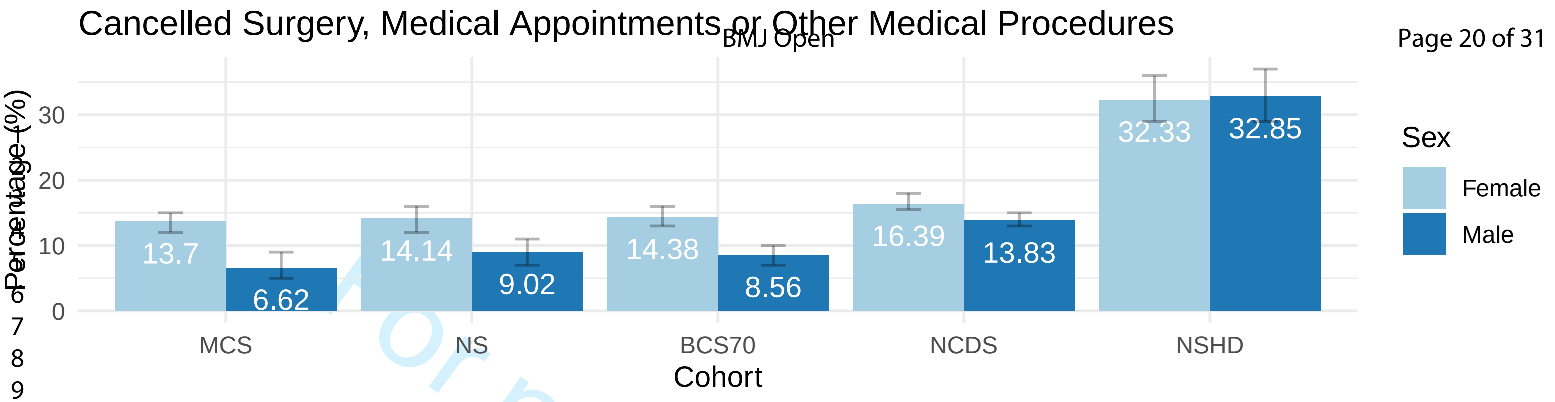

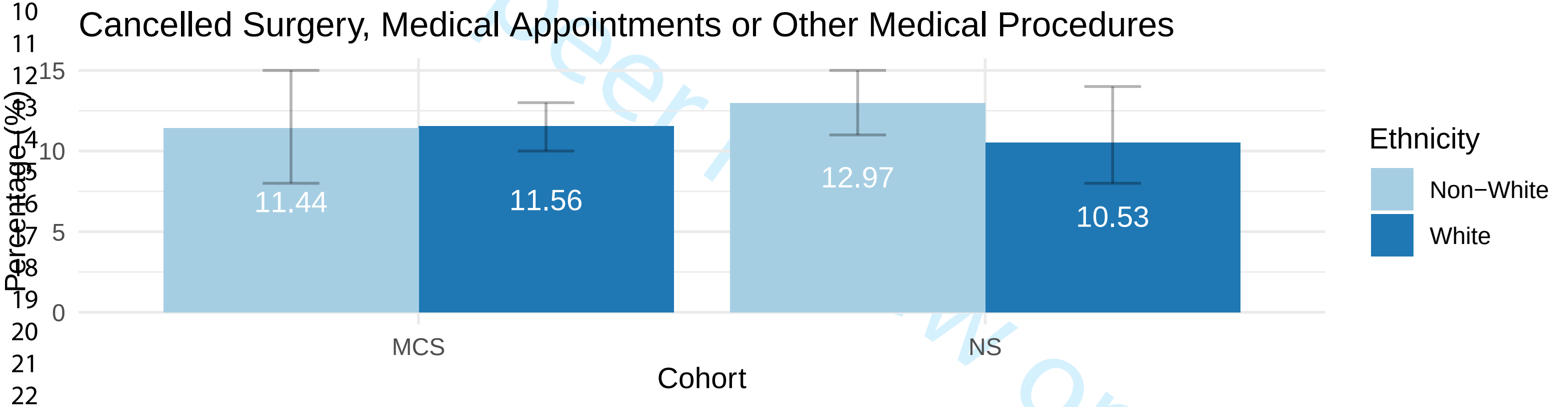

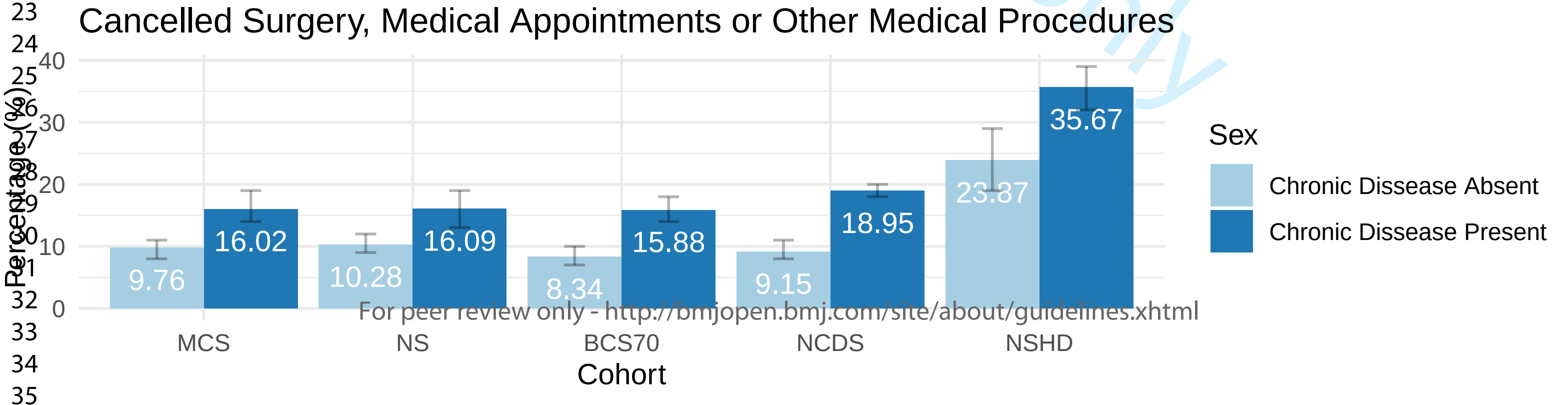

For peer review only

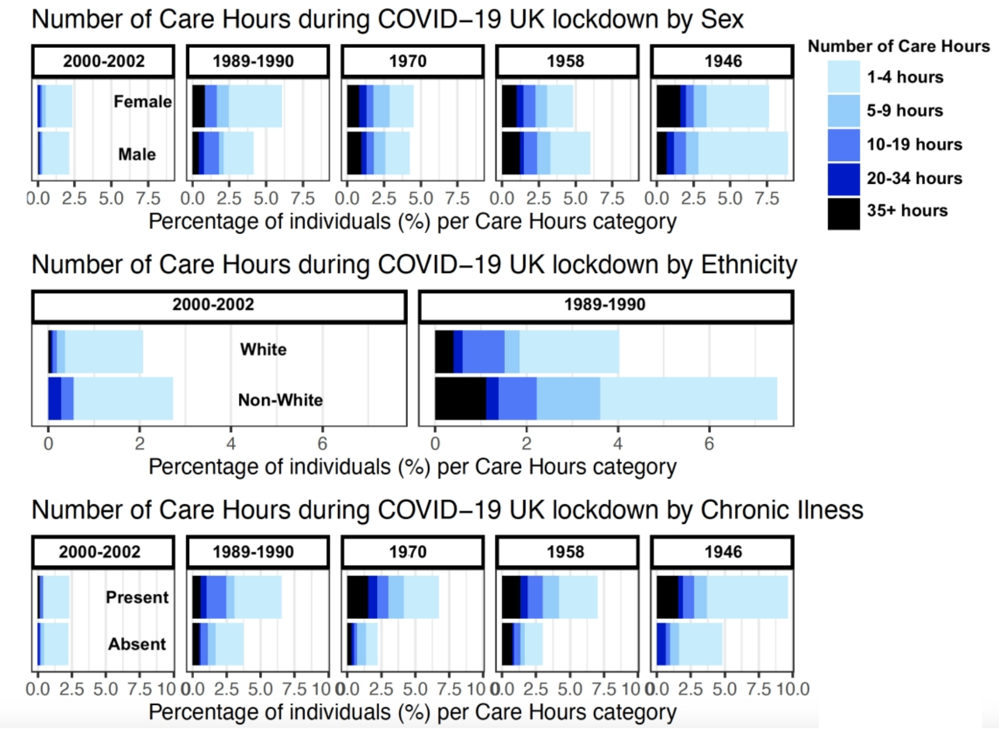

Figure 2. Bar charts illustrating the percentage of participants requiring support based on the number of care hours needed during the UK COVID-19 national lockdown stratified by sex, ethnicity and the presence of chronic illness across the cohorts.

SUPPLEMENTARY MATERIAL FOR:

Inequality of access to health and care services during lockdown–Findings from the COVID-19 survey in five UK national longitudinal studies

Constantin-Cristian Topriceanu[1,2], Andy Wong[2], James C Moon[3,4], Alun D Hughes[1,2], David Bann[5], Nish Chaturvedi[1,2], Praveetha Patalay[2,5], Gabriella Conti[6], Gabriella Captur[1,2,7]

Author Affiliations:
1. UCL Medical School, 74 Huntley St, Bloomsbury, London WC1E 6DE
2. UCL MRC Unit for Lifelong Health and Ageing, University College London, Fitzrovia, London WC1E 7HB
3. UCL Institute of Cardiovascular Science, University College London, Gower Street, London WC1E 6BT, UK
4. Cardiac MRI Unit, Barts Heart Centre, West Smithfield, London, EC1A 7BE
5. Centre for Longitudinal Studies, Department of Social Science, University College London, London WC1H 0AL, UK
6. Department of Economics, University College London, London WC1 H0AX, UK
7. The Royal Free Hospital, Centre for Inherited Heart Muscle Conditions, Cardiology Department, Pond Street, Hampstead, London NW3 2QG, UK

For peer review only

Supplementary Table S1. Associations of sex with cancelled surgery, medical procedures or other medical appointments during lockdown after adjustment for children under 16 in the household.

Cohort study birth year	Sex^	
	OR (95% CI)	*p*-value
**1946** (*n*=1154)	0·98 (0·76, 1·25)	0·839
**1958** (*n*=4940)	1·18 (1·01, 1·39)	**0·040**
**1970** (*n*=4008)	1·78 (1·45, 2·21)	**<0·0001**
**1989-1990** (*n*=1796)	1·66 (1·20, 2·34)	**0·003**
**2000-2002** (*n*=2510)	2·29 (1·66, 3·25)	**<0·0001**

Sex was coded as 0=male and 1=female.
All analyses used generalized linear models with logit link. Significant *p*-values are highlighted in bold.
^Adjustment was made for non-response weight, shielding letter and children under 16 in the household.
1946, refers to National Study of Health and Development (NSHD); 1958, refers to National Child Development Study (NCDS); 1970, refers to British Cohort Study (BCS70); 1989-1990, refers to Next Steps (NS); 2000-2002, refers to Millennium Cohort Study (MCS); CI, confidence interval; OR, odds ratio.

Supplementary Table S2. Associations of sex with cancelled surgery, medical procedures or other medical appointments during lockdown after adjustment for psychological distress.

Cohort study birth year	Sex^	
	OR (95% CI)	*p*-value
**1946** (*n*=1059)	1·00 (0·77, 1·30)	0·983
**1958** (*n*=4612)	1·12 (0·95, 1·32)	0·191
**1970** (*n*=3481)	1·82 (1·34, 2·30)	**<0·0001**
**1989-1990** (*n*=1697)	1·71 (1·23, 2·44)	**0·002**
**2000-2002** (*n*=2461)	2·18 (1·55, 3·11)	**<0·0001**

Sex was coded as 0=male and 1=female.
All analyses used generalized linear models with logit link. Significant *p*-values are highlighted in bold.
^Adjustment was made for non-response weight, shielding letter and psychological distress.
Abbreviations as in ***Supplementary Table S1***.

Supplementary Table S3. Associations of sex with cancelled surgery, medical procedures or other medical appointments during lockdown after adjustment for chronic illness.

Cohort study birth year	Sex^	
	OR (95% CI)	*p*-value
**1946** (*n*=1121)	1·01 (0·79, 1·30)	0·822
**1958** (*n*=5015)	1·24 (1·06, 1·46)	**0·007**
**1970** (*n*=4037)	1·79 (1·45, 2·21)	**<0·0001**
**1989-1990** (*n*=1800)	1·64 (1·19, 2·30)	**0·003**
**2000-2002** (*n*=2487)	2·08 (1·51, 2·93)	**<0·0001**

Sex was coded as 0=male and 1=female.
All analyses used generalized linear models with logit link. Significant *p*-values are highlighted in bold.
^Adjustment was made for non-response weight, shielding letter and chronic illness.
Abbreviations as in ***Supplementary Table S1***.

For peer review only

Supplementary Table S4. Meta-regression for the effect of age on cancelled surgery, medical procedures or other medical appointments and number of care hours needed during lockdown.

**Analysis**	**Outcome**	***p*-value^**
Cancelled appointments	Sex	0·196
	Chronic Illness	0·928
Number of care hours	Ethnicity*	N/A
	Chronic Illness	0·352

^p-values for the test of moderators for the restricted cubic spline model are provided.
*Meta-regression was not feasible as only 2 studies recorded ethnicity.
N/A, not available.

Supplementary Table S5. Associations of ethnicity with the number of care hours during lockdown after adjustment for keyworker status.

**Cohort study birth year**	**Ethnicity^**	
	**OR (95% CI)**	***p*-value**
**1989-1990** (*n*=1785)	0·45 (0·28, 0·75)	**0·002**
**2000-2002** (*n*=2586)	0·76 (0·40, 1·63)	0·453

Ethnicity was coded as 0=non-White and 1=White.
All analyses used generalized linear models with logit link. Significant *p*-values are highlighted in bold.
^Adjustment was made for non-response weight, shielding letter, sex and keyworker status.
*Abbreviations as in **Supplementary Table S1**.*

Supplementary Table S6. Associations of sex and with cancelled surgery, medical procedures or other medical appointments during lockdown after multiple imputation.

**Cohort study birth year**	**Sex^**	
	**OR (95% CI)**	***p*-value**
**1958** (*n*=18558)	1·19 (1·08, 1·30)	**<0·0001**
**1970** (*n*=19006)	1·83 (1·61, 2·07)	**<0·0001**
**1989-1990** (*n*=16122)	1·50 (1·23, 1·83)	**0·002**
**2000-2002** (*n*=22131)	1·75 (1·32, 2·33)	**0·003**

Sex was coded as 0=male and 1=female.
All analyses used generalized linear models with logit link. Significant *p*-values are highlighted in bold.
^ Adjustment was made for shielding letter.
*Abbreviations as in **Supplementary Table S1**.*

For peer review only

Supplementary Table S7. Association of sex, ethnicity, socio-economic position and the presence of chronic illness with number of care hours during lockdown after further adjustment for hours of care before lockdown.

Cohort study birth year	Sex^			Ethnicity*			Socio-economic position†			Chronic Illness§		
	**OR (95% CI)**	***p*-value**	**Brant test *p*-value**	**OR (95% CI)**	***p*-value**	**Brant test *p*-value**	**OR (95% CI)**	***p*-value**	**Brant test *p*-value**	**OR (95% CI)**	***p*-value**	**Brant test *p*-value**
**1946** (*n*=1170)	1·25 (0·76, 2·07)	0·377	**<0·0001**	N/A	N/A	N/A	0·85 (0·35, 2·06)	0·718	0·148	1·93 (1·02, 4·00)	**0·048**	0·260
**1958** (*n*=4860)	1·42 (1·02, 1·99)	**0·038**	**0·004**	N/A	N/A	N/A	1·22 (0·66, 2·26)	0·523	**0·046**	1·59 (1·05, 2·45)	**0·031**	**0·015**
**1970** (*n*=3960)	0·91 (0·61, 1·37)	0·654	**<0·0001**	N/A	N/A	N/A	1·37 (0·67, 2·84)	0·389	**<0·0001**	1·75 (1·10, 2·81)	**0·020**	**<0·0001**
**1989-1990** (*n*=1782)	0·60 (0·33, 1·10)	0·097	0·153	0·55 (0·29, 1·11)	0·086	**<0·0001**	1·34 (0·48, 3·83)	0·457	**<0·0001**	1·61 (0·87, 2·98)	0·128	**<0·0001**
**2000-2002** (*n*=2478)	0·95 (0·51, 1·81)	0·868	0·967	0·70 (0·32, 1·76)	0·404	1·00	2·18 (0·76, 6·42)	0·148	0·998	1·21 (0·62, 2·28)	0·562	0·938

Sex was coded as 0=male and 1=female and ethnicity as 0=non-White and 1=White· Socio-economic was coded using childhood social class from 1=managerial to 6=unskilled, but ridit scores were used. Chronic illness was coded as 0=absent and 1=present.
All analyses used generalized linear models with ordinal logit link. Significant *p*-values are highlighted in bold.
^ Adjustment was made for survey non-response weight, previous care hours and shielding letter.
* Adjustment was made for survey non-response weight, previous care hours, shielding letter and sex.
† Adjustment was made for survey non-response weight, previous care hours, shielding letter, sex and ethnicity.
§ Adjustment was made for survey non-response weight, previous care hours shielding letter, sex, ethnicity and SEP.
*N/A, not available. Other abbreviations as in **Supplementary Table S1**.*

For peer review only

Supplementary Table S8. **Association of sex, ethnicity, socio-economic position and the presence of chronic illness with number of care hours during lockdown using multinomial logistic regression.**

	**Sex^**			**Ethnicity***			**Socio-economic position†**			**Chronic Illness§**		
**Cohort study birth year**	**Neverø**	**Low RRR ⸍ (95% CI)**	**High RRR ˃ (95% CI)**	**Never**	**Low RRR (95% CI)**	**High RRR (95% CI)**	**Never**	**Low RRR (95% CI)**	**High RRR (95% CI)**	**Never**	**Low RRR (95% CI)**	**High RRR (95% CI)**
**1946** (*n*=1170)	ref	1·30 (0·79, 2·17)	0·82 (0·38, 1·81)	N/A	N/A	N/A	ref	0·93 (0·39, 2·19)	2·18 (0·55, 8·70)	ref	1·90 (0·97, 3·72)	3·15 (0·93, 10·28)
**1958** (*n*=4884)	ref	1·34 (0·67, 1·50)	1·01 (0·94, 1·92)	N/A	N/A	N/A	ref	1·34 (0·94, 1·91)	1·24 (0·62, 2·48)	ref	**2·27 (1·46, 3·53)**	**2·08 (1·27, 3·40)**
**1970** (*n*=3972)	ref	1·02 (0·66, 1·60)	1·27 (0·72, 2·23)	N/A	N/A	N/A	ref	0·74 (0·35, 1·57)	0·69 (0·27, 1·77)	ref	**2·07 (1·28, 3·36)**	**4·39 (2·18, 8·84)**
**1989-1990** (*n*=1787)	ref	0·49 (0·27, 0·89)	1·11 (0·49, 2·51)	ref	**0·73 (0·20, 0·70)**	0·37 (0·27, 1·99)	ref	0·64 (0·24, 1·76)	1·41 (0·36, 5·43)	ref	1·53 (0·84, 2·78)	1·83 (0·83, 4·05)
**2000-2002** (*n*=2605)	ref	0·50 (0·44, 1·84)	0·90 (0·01, 3·53)	ref	0·92 (0·01, 1·6)	0·19 (0·36, 2·36)	ref	1·98 (0·19, 11·16)	4·62 (0·66, 5·97)	ref	1·08 (0·55, 2·12)	**9·24 (1·03, 83·25)**

Sex was coded as 0=male and 1=female and ethnicity as 0=non-White and 1=White· Socio-economic was coded using childhood social class from 1=managerial to 6=unskilled, but ridit scores were used. Chronic illness was coded as 0=absent and 1=present.
All analyses used multinomial logistic regression.
Statistically significant findings (95% CI exclude the null value) are highlighted in bold.
^ Adjustment was made for survey non-response weight and shielding letter.
* Adjustment was made for survey non-response weight, shielding letter and sex.
† Adjustment was made for survey non-response weight, shielding letter, sex and ethnicity.
§ Adjustment was made for survey non-response weight shielding letter, sex, ethnicity and SEP.
ø Defined as 0 hours of care needed post-COVID-19.
⸍ Defined as 1-9 hours of care needed post-COVID-19.
˃ Defined as 10+ hours of care needed post-COVID-19.
*ref, reference; RRR, relative risk ratio. Other abbreviations as in **Supplementary Table S1**.*

For peer review only

For peer review only - http://bmjopen.bmj.com/site/about/guidelines.xhtml

Supplementary Table S9. Association of socio-economic position surrogates with cancelled surgery, medical procedures or other medical appointments during lockdown.

Cohort study birth year	SEP as highest educational attainment ridit score^			SEP as pre-COVID financial difficulties ridit score^		
	n	**OR (95% CI)**	***p*-value**	***n***	**OR (95% CI)**	***p*-value**
**1946**	1089	1·43 (0·92, 2·21)	0·111	1145	1·28 (0·84, 1·97)	0·246
**1958**	4972	1·09 (0·83, 1·42)	0·559	4878	1·11 (0·85, 1·46)	0·454
**1970**	3850	1·12 (0·80, 1·58)	0·501	3978	1·10 (0·79, 1·54)	0·576
**1989-1990**	1722	1·41 (0·84, 2·37)	0·190	1768	1·49 (0·91, 2·44)	0·119
**2000-2002**	2444	0·84 (0·54, 1·31)	0·477	2413	0·90 (0·58, 1·40)	0·654

All analyses used generalized linear models with logit link.
^ Adjustment was made for survey non-response weight, shielding letter, sex and ethnicity.

For peer review only

Supplementary Table S10. Association of socio-economic position surrogates with the number of care hours during lockdown.

**Cohort study birth year**	**SEP as highest educational attainment ridit score^**			**SEP as pre-COVID financial difficulties ridit score^**		
	n	**OR (95% CI)**	***p*-value**	***n***	**OR (95% CI)**	***p*-value**
**1946**	1170	1·17 (0·77, 1·79)	0·672	1170	1·18 (0·58, 2·40)	0.657
**1958**	4789	1·12 (0·72, 1·75)	0·609	4836	1·20 (0·77, 1·87)	0·407
**1970**	3747	0·68 (0·39, 1·20)	0·182	3954	0·81 (0·47, 1·39)	0·345
**1989-1990**	1666	0·99 (0·43, 2·25)	0·979	1768	0·92 (0·43, 2·00)	0·841
**2000-2002**	2444	1·53 (0·57, 4·14)	0·408	2420	1·86 (0·73, 4·79)	0·196

All analyses used generalized linear models with ordinal logit link·
^ Adjustment was made for survey non-response weight, shielding letter, sex and ethnicity.
Abbreviations as in ***Supplementary Table S1***.

For peer review only

STROBE CHECKLIST FOR:

Inequality of access to health and care services during lockdown–Findings from the COVID-19 survey in five UK national longitudinal studies

Constantin-Cristian Topriceanu[1,2], Andy Wong[2], James C Moon[3,4], Alun D Hughes[1,2], David Bann[5], Nish Chaturvedi[1,2], Praveetha Patalay[2,5], Gabriella Conti[6], Gabriella Captur[1,2,7]

Author Affiliations:

1. UCL Medical School, 74 Huntley St, Bloomsbury, London WC1E 6DE
2. UCL MRC Unit for Lifelong Health and Ageing, University College London, Fitzrovia, London WC1E 7HB
3. UCL Institute of Cardiovascular Science, University College London, Gower Street, London WC1E 6BT, UK
4. Cardiac MRI Unit, Barts Heart Centre, West Smithfield, London, EC1A 7BE
5. Centre for Longitudinal Studies, Department of Social Science, University College London, London WC1H 0AL, UK
6. Department of Economics, University College London, London WC1 H0AX, UK
7. The Royal Free Hospital, Centre for Inherited Heart Muscle Conditions, Cardiology Department, Pond Street, Hampstead, London NW3 2QG, UK

For peer review only

STROBE Statement—Checklist of items that should be included in reports of ***cohort studies***

	Item No	Recommendation	Page
**Title and abstract**	1	(*a*) Indicate the study's design with a commonly used term in the title or the abstract	**1**
		(*b*) Provide in the abstract an informative and balanced summary of what was done and what was found	**2**
**Introduction**			
Background/rationale	2	Explain the scientific background and rationale for the investigation being reported	**4**
Objectives	3	State specific objectives, including any prespecified hypotheses	**4**
**Methods**			
Study design	4	Present key elements of study design early in the paper	**4**
Setting	5	Describe the setting, locations, and relevant dates, including periods of recruitment, exposure, follow-up, and data collection	**4**
Participants	6	(*a*) Give the eligibility criteria, and the sources and methods of selection of participants. Describe methods of follow-up	**5**
		(*b*) For matched studies, give matching criteria and number of exposed and unexposed	**Yes**
Variables	7	Clearly define all outcomes, exposures, predictors, potential confounders, and effect modifiers. Give diagnostic criteria, if applicable	**5**
Data sources/ measurement	8*	For each variable of interest, give sources of data and details of methods of assessment (measurement). Describe comparability of assessment methods if there is more than one group	**5**
Bias	9	Describe any efforts to address potential sources of bias	**4,5,6**
Study size	10	Explain how the study size was arrived at	**5,6**
Quantitative variables	11	Explain how quantitative variables were handled in the analyses. If applicable, describe which groupings were chosen and why	**5,6**
Statistical methods	12	(*a*) Describe all statistical methods, including those used to control for confounding	**5,6**
		(*b*) Describe any methods used to examine subgroups and interactions	**5,6**
		(*c*) Explain how missing data were addressed	**5,6**
		(*d*) If applicable, explain how loss to follow-up was addressed	**5,6**
		(*e*) Describe any sensitivity analyses	**5,6**
**Results**			
Participants	13*	(a) Report numbers of individuals at each stage of study—eg numbers potentially eligible, examined for eligibility, confirmed eligible, included in the study, completing follow-up, and analysed	**7**
		(b) Give reasons for non-participation at each stage	**7**
		(c) Consider use of a flow diagram	**N/A**
Descriptive data	14*	(a) Give characteristics of study participants (eg demographic, clinical, social) and information on exposures and potential confounders	**7**
		(b) Indicate number of participants with missing data for each variable of interest	**7**
		(c) Summarise follow-up time (eg, average and total amount)	**4**
Outcome data	15*	Report numbers of outcome events or summary measures over time	**7**

For peer review only

Main results	16	(*a*) Give unadjusted estimates and, if applicable, confounder-adjusted estimates and their precision (eg, 95% confidence interval). Make clear which confounders were adjusted for and why they were included	**7**
		(*b*) Report category boundaries when continuous variables were categorized	**7**
		(*c*) If relevant, consider translating estimates of relative risk into absolute risk for a meaningful time period	**N/A**
Other analyses	17	Report other analyses done—eg analyses of subgroups and interactions, and sensitivity analyses	**8**
**Discussion**			
Key results	18	Summarise key results with reference to study objectives	**9**
Limitations	19	Discuss limitations of the study, taking into account sources of potential bias or imprecision. Discuss both direction and magnitude of any potential bias	**10**
Interpretation	20	Give a cautious overall interpretation of results considering objectives, limitations, multiplicity of analyses, results from similar studies, and other relevant evidence	**9**
Generalisability	21	Discuss the generalisability (external validity) of the study results	**9.10**
**Other information**			
Funding	22	Give the source of funding and the role of the funders for the present study and, if applicable, for the original study on which the present article is based	**10,11**

*Give information separately for exposed and unexposed groups.

For peer review only

BMJ Open

Evaluating access to health and care services during lockdown by the COVID-19 survey in five UK national longitudinal studies

Journal:	*BMJ Open*
Manuscript ID	bmjopen-2020-045813.R1
Article Type:	Original research
Date Submitted by the Author:	04-Feb-2021

Complete List of Authors:	Topriceanu, Constantin-Cristian; University College London,<br>Wong, Andrew; UCL, MRC Unit for Lifelong Ageing<br>Moon, James; Barts Heart Centre, Cardiac Imaging Department<br>Hughes, A; University College London<br>Bann, David; University College London,<br>Chaturvedi, Nishi; University College London,<br>Patalay, Praveetha ; UCL<br>Conti, Gabriella; UCL<br>Captur, Gaby; University College London

**Primary Subject Heading**:	Epidemiology
Secondary Subject Heading:	Health policy, Health services research, Public health
Keywords:	EPIDEMIOLOGY, Quality in health care < HEALTH SERVICES ADMINISTRATION & MANAGEMENT, PUBLIC HEALTH

SCHOLARONE™
Manuscripts

I, the Submitting Author has the right to grant and does grant on behalf of all authors of the Work (as defined in the below author licence), an exclusive licence and/or a non-exclusive licence for contributions from authors who are: i) UK Crown employees; ii) where BMJ has agreed a CC-BY licence shall apply, and/or iii) in accordance with the terms applicable for US Federal Government officers or employees acting as part of their official duties; on a worldwide, perpetual, irrevocable, royalty-free basis to BMJ Publishing Group Ltd ("BMJ") its licensees and where the relevant Journal is co-owned by BMJ to the co-owners of the Journal, to publish the Work in this journal and any other BMJ products and to exploit all rights, as set out in our licence.

The Submitting Author accepts and understands that any supply made under these terms is made by BMJ to the Submitting Author unless you are acting as an employee on behalf of your employer or a postgraduate student of an affiliated institution which is paying any applicable article publishing charge ("APC") for Open Access articles. Where the Submitting Author wishes to make the Work available on an Open Access basis (and intends to pay the relevant APC), the terms of reuse of such Open Access shall be governed by a Creative Commons licence – details of these licences and which Creative Commons licence will apply to this Work are set out in our licence referred to above.

Other than as permitted in any relevant BMJ Author's Self Archiving Policies, I confirm this Work has not been accepted for publication elsewhere, is not being considered for publication elsewhere and does not duplicate material already published. I confirm all authors consent to publication of this Work and authorise the granting of this licence.

For peer review only

Evaluating access to health and care services during lockdown by the COVID-19 survey in five UK national longitudinal studies

Constantin-Cristian Topriceanu[1,2], Andrew Wong[2], James C Moon[3,4], Alun D Hughes[1,2], David Bann[5], Nishi Chaturvedi[1,2], Praveetha Patalay[2,5], Gabriella Conti[6], Gaby Captur[1,2,7]

Author Affiliations:
1. UCL Medical School, 74 Huntley St, Bloomsbury, London WC1E 6DE
2. UCL MRC Unit for Lifelong Health and Ageing, University College London, London WC1E 7HB
3. UCL Institute of Cardiovascular Science, University College London, Gower Street, London WC1E 6BT, UK
4. Cardiac MRI Unit, Barts Heart Center, West Smithfield, London, EC1A 7BE
5. Center for Longitudinal Studies, Department of Social Science, University College London, London WC1H 0AL, UK
6. Department of Economics and UCL Social Research Institute, University College London, London WC1 H0AX, UK
7. The Royal Free Hospital, Center for Inherited Heart Muscle Conditions, Cardiology Department, Pond Street, Hampstead, London NW3 2QG, UK

Corresponding author:
Gabriella Captur
Consultant Cardiologist in Inherited Heart Muscle Conditions,
Senior Clinical Lecturer,
MRC Unit for Lifelong Health and Ageing at University College London,
1-19 Torrington Place,
Fitzrovia, London WC1E 7HB, UK
E-mail: gabriella.captur@ucl.ac.uk, Phone No: +44 207460059

For peer review only

ABSTRACT

Objective: Access to health services and adequate care is influenced by sex, ethnicity, socio-economic position (SEP) and burden of co-morbidities. Our study aimed to assess whether the COVID-19 pandemic further deepened these already existing health inequalities.

Design: Cross-sectional study.

Setting: Data was collected from five longitudinal age-homogenous British cohorts (born in 2001, 1990, 1970, 1958 and 1946).

Participants: A web and telephone-based survey was sent to the cohorts. Anybody who responded to the survey was included resulting in 14891 eligible participants.

Main outcomes measured: The survey provided data on cancelled surgical or medical appointments, and the number of care hours received during the UK COVID-19 national lockdown.

Interventions: Using binary or ordered logistic regression, we evaluated whether these outcomes differed by sex, ethnicity, SEP and having a chronic illness. Adjustment was made for study-design, non-response weights, psychological distress, presence of children or adolescents in the household, keyworker status, and whether participants had received a shielding letter. Meta-analyses were performed across the cohorts and meta-regression evaluated the effect of age as a moderator.

Results: Females (OR 1·40, 95% confidence interval [1·27,1·55]) and those with a chronic illness (OR 1·84 [1·65-2·05]) experienced significantly more cancellations during lockdown (all $p<0{\cdot}0001$). Ethnic minorities and those with a chronic illness required a higher number of care hours during the lockdown (both OR ≈2·00, all $p<0{\cdot}002$). Age was not independently associated with either outcome in meta-regression. SEP was not associated with cancellation or care hours.

Conclusions: The UK government’s lockdown approach during the COVID-19 pandemic appears to have deepened existing health inequalities, impacting predominantly females, ethnic-minorities and those with chronic illnesses. Public health authorities need to implement urgent policies to ensure equitable access to health and care for all in preparation for a third wave.

Keywords: access to healthcare; health inequalities, SARS-CoV2 pandemic.

For peer review only - http://bmjopen.bmj.com/site/about/guidelines.xhtml

ARTICLE SUMMARY

Strengths and limitations of this study

- A strength of the study is the implicit age homogeneity of birth cohort participants. This enables age-matching within each cohort for each data collection sweep.

- Combining five cohorts spanning multiple age groups (19, 30, 50, 62 and 74 years old) enabled a better understanding of how the COVID-19 pandemic has affected different generations.

- The longitudinal nature of the cohorts enabled the derivation of individualized non-response weights for each of the participants which have been included in all analyses. This enabled us to address non-response bias rendering results more generalizable.

- As self-reported measures were used, the number of care hours needed before and during lockdown were subject to reporting biases. In addition, single categorical outcome variables do not have the capacity to measure the impact spectrum generated by the cancelled appointments as well as the reduced number of care hours received.

- We binarized the ethnicity variable to enable sufficient sample sizes for comparisons, but this precluded more detailed comparisons between the diverse ethnic groups which exist in the UK. Older cohorts (0, 62 and 74 years old) consist of almost only white participants, so we were unable to describe findings for older persons from minority ethnic groups that may have been most adversely affected by lockdown. Our younger cohorts (19 and 30 years old) include less than 20% non-White participants, which could result in specious associations. Our analysis did not take into account racism as a structural actor to explain the disparities observed and further work will be needed in future studies to address.

For peer review only

INTRODUCTION

On 11 March 2020, the World Health Organization declared the novel severe acute respiratory syndrome-coronavirus-19 (SARS-CoV-2, also known as COVID-19) outbreak a global pandemic. As the United Kingdom (UK) was facing a surge of new cases, the government-imposed lockdown restrictions across England, Scotland and Wales on 23 March 2020 in order to limit the spread of the virus. Although the restrictions were gradually relaxed, the most widely accepted end-date of the lockdown is considered to be the 4th of July 2020 when non-essential businesses such as bars, restaurants opened. Delivery of routine care across the UK National Health Service was hampered by the pandemic crisis and the lockdown.

Access to health services and adequate care has previously been shown to be influenced by sex, ethnicity, socio-economic position (SEP) and burden of co-morbidities[1 2]. However, it is unknown whether access to health and care services during the COVID-19 pandemic differed by these factors, potentially further widening already existing health inequalities[3]. Evidence from previous pandemics suggests this possibility, but data is missing in the context of COVID-19 currently. To answer these questions, a web-based survey was sent to participants in five UK national longitudinal studies, spanning multiple generations; data were collected during the core UK lockdown, between 2nd of May and 1st of June 2020. The survey questions can be accessed here: https://cls.ucl.ac.uk/wp-content/uploads/2020/12/COVID-19-Online-Survey-Questionnaire-Wave-1-April-2020-Version-2.pdf." We investigated the number of participants having a cancelled surgical or medical appointment and the number of care hours received for self or other household members over a week during the lockdown. We analyzed how these outcomes varied by already established factors contributing to health inequalities. The importance of cancellations stems from the potential consequences of healthcare deprivation, while the number of weekly care hours has been shown to predict admission to long-term care facilities especially in the older population[4].

METHODS

Study design

The five UK national longitudinal studies were: National Study of Health and Development Study (NSHD)[5], National Child Development Study (NCDS)[6], 1970 British Cohort Study (BCS70)[7], Next Steps (NS)[8] and Millennium Cohort Study (MCS)[9]. NSHD participants were born in 1946, NCDC in 1958, BCS70 in 1970 and MCS in 2000-2002 and all participants were followed-up from birth (all birth cohorts), while NS is a longitudinal cohort study whose participants, born in 1989-1990, have been followed-up from adolescence. The cohorts have been extensively followed up with periodic assessments which have been described elsewhere. During the COVID-19 pandemic (May 2020), an online questionnaire was sent to each participant from each cohort. The questionnaire was designed to explore the physical health, health behaviours, social contact and support, loneliness and mental health, household relationships and care needs, housing situation, employment, finances and benefits and education during the height of the COVID-19 pandemic. The questionnaire format was mostly multiple choice with either binary (yes/no) or categorical response options. Towards the end of the survey, participants also had the option to enter free text to describe their particular

For peer review only - http://bmjopen.bmj.com/site/about/guidelines.xhtml

COVID-19 experience. There were no inceptives, just an invite to participate sent by email. Two email reminders were sent to NCDS, BCS70, NS and MCS participants who had not started, or who had partially completed the survey. A single email reminder was sent to NSHD participants. Cohort participants contributed to the study design. However, it was not appropriate or possible to involve participants or the public in the conduct, or reporting, or dissemination plans of our research.

Ethical approval

Ethical approval was obtained from the relevant committees and from the University College London/Institute of Education research ethics committee (REC1334). All participants gave informed consent before taking part.

Patient and public involvement

It was not appropriate or possible to involve the study participants or the public in the design or conduct of our research. However, we plan to disseminate the results to the study participants.

Outcomes

Individuals experiencing a healthcare-related cancellation in the form of a cancelled surgery, medical procedure or other medical appointment at any time since the beginning of the SARS-CoV-2 pandemic were scored as 1, or 0 otherwise. The number of hours of help received for self or other household member in a typical week during lockdown was recorded in six categories: 0, 1-4, 5-9, 10-19, 20-34 or 35+ hours. The variable encompasses both home healthcare and social help. The care provider could be a family member, a friend, a professional paid carer or voluntary helper. Thus, we had two outcomes: cancelled surgery, medical procedures or other medical appointments and the number of hours of help received in a week.

Exposures

Sex was recoded as 0=male and 1=female, while ethnicity was recoded as 0=non-White and 1=White. As NSHD, NCDS and BCS70 consist mostly of White participants, ethnicity data was examined only for the NS and MCS cohorts. Highest educational attainment and financial difficulties prior to COVID-19 were used as a proxy for adult SEP. Highest educational attainment was categorized as: degree/higher, advanced-level exam/diploma, ordinary-level exam/general certificate of secondary education or none. Financial difficulties before lockdown were self-rated using the following options: managing comfortably, all right, getting by and difficult. As many MCS participants were still undertaking education and financially dependent on their families, their parents' highest education and financial difficulties were used. Childhood social class has also been recorded according to the UK Office of Population Censuses and Surveys Registrar General's social class resulting in six categories: professional, managerial and technical, skilled non-manual, skilled manual, partly-skilled and unskilled. Participants were asked to report whether they had a long-standing illness (yes/no). In addition, the nature of each chronic illness was also broadly recorded. Thus, our exposures were sex, ethnicity, SEP and the presence of a chronic illness.

For peer review only - http://bmjopen.bmj.com/site/about/guidelines.xhtml

Covariates

Participants that were clinically extremely vulnerable and at high-risk of complications from potential SARS-CoV-2 infection were sent a letter or text message from the National Health Service (NHS) or Chief Medical Officer advising them to shield, that is, to stay at home except for specific purposes and avoid contact with persons they do not live with, except for specific purposes. Receipt of such a shielding letter was recorded as a binary variable (yes/no). The number of hours of help received for self or another household member in a week before the pandemic was recorded as above.

Women are more likely to care for children aged less than 16 years and more likely to report psychological distress [10]. Thus, sex differences were further explored using the two covariates. The presence of children aged less than 16 years in the household as well as the self-reported presence of psychological distress during lockdown were recorded (yes/no). Psychological distress was measured using the General Health Questionnaire (GHQ-12[11]) defined as 0 if <4, and 1 if ≥4 for NSHD and NS, and using a shortened 9-item Malaise inventory defined as 0 if <4, and 1 if ≥4 for NCDS and BCS70 as previously described[12]. For MCS, the Kessler K6 score was used defined as 1 if ≥13 and 0 otherwise[13].

In the literature, non-white ethnicity was associated with key-worker status[14] and COVD-19 infection[15]. Thus, ethnicity differences were further explored using the two covariates. Keyworker status was self-rated based on whether participants' work was classified as critical to the COVID-19 response. COVID-19 infection was recoded as 0=no and 1=yes, based on a positive antigen or antibody test or strong personal suspicion due to symptoms.

Statistical analysis

Statistical analysis was performed in R (version-3.6.0). Frequency distribution of continuous data were assessed visually using histograms. Categorical variables were expressed as counts and percent for each available category. Within each cohort, childhood SEP, highest educational attainment and financial difficulties were converted into cumulative rank probabilities (ridit scores) to quantify the difference in outcomes comparing the lowest with highest SEP (i.e., the relative indices of inequality)[16]. Models containing all socio-economic variables were assessed for multicollinearity via the variance inflation method. As childhood SEP was multicollinear with the other two, had the least amount of missing data, and as it could impact on adult behaviors and health outcomes independently of adult SEP[17], it was used in subsequent analysis. However, we additionally report the results from the analyses using SEP based on highest educational attainment, and financial difficulties respectively.

Separate regression models were using sex, ethnicity, SEP and presence of chronic illness as predictors of cancelled appointments or number of care hours needed during lockdown. Generalized linear models with logit link were employed to predict cancelled appointments, while ordinal logistic regression was used to predict the number of care hours needed. The proportional odds assumption for ordinal logistic regression was tested using a Brant test[18].Weights to account for the stratified survey designs of 1946,

For peer review only - http://bmjopen.bmj.com/site/about/guidelines.xhtml

1990 and 2000-2002 cohorts[19] have been previously developed. Logistic regression models predicting each participant's response during the COVID-19 data sweep based on known demographic, socioeconomic, household and individual predictors of non-response at previous data collection points were used to calculate non-response weights[20 21]. In the logistic regression models, missing covariate values were generated using multiple imputation. For each COVID-19 survey respondent, the probability of response was calculated, and non-response weights were derived as the inverse probability of response with further calibration to sum to the number of respondents in each cohort. The stratified survey design and non-response weights were combined to generate a combined weight[16]. An individualized combined weight was derived for each study respondent (full details available in the Centre for Longitudinal Study COVID-19 Survey User Guide: https://cls.ucl.ac.uk/wp-content/uploads/2020/12/UCL-Cohorts-COVID-19-Survey-user-guide.pdf). Predictors were included sequentially one at a time. Sex analyses were adjusted for these individualized combined weights and for receipt of a shielding letter. All other analyses were similarly adjusted, but ethnicity analyses were additionally adjusted for sex; SEP analyses additionally adjusted for sex and ethnicity; and chronic illness analyses additionally adjusted for sex, ethnicity and SEP.

Gender differences were further evaluated by adjusting for children less than 16 years in the household and psychological distress during the lockdown. As females are more likely to have a chronic disease, gender differences were also evaluated after adjustment for the presence of a chronic disease[22]. Ethnicity differences were further explored by adjusting for key worker status as ethnic minorities have been reported to be over-represented as key workers in the literature[14]. As other studies have shown greater COVID-19 positivity rates among ethnic minorities[15], further adjustment for COVID-19 infection was pursued for ethnicity in all analyses.

We also explored whether individuals with multiple co-morbidities (defined as 2 or more) were more likely to experience a cancelled appointment or require a higher number of care hours compared to individuals with a single long-standing illness. Comparisons were made using Chi-squared test for the cancelled appointment analyses and Mann-Whitney U test for the care hours analyses.

Cohort-specific analyses were conducted initially. Meta-analyses were then performed across the cohorts, only if there was a significant result in at least one of the cohorts. Heterogeneity was evaluated using Cochran' Q test and I^2 statistic. As smaller samples have more sampling errors in their effect estimate, larger effect size might emerge[23]. Thus, funnel plot asymmetry was evaluated using Egger's test. Meta-regression was conducted with age/cohort as a moderator in order to determine whether it was a source of heterogeneity. As the associations between age and our outcomes are likely to be non-linear based on visual inspection, we performed the meta-regression using restricted cubic splines modelling[24].

We ran sensitivity analyses in which we: (1) simulated a complete case analysis through multiple imputation to verify the reliability of observed sex-related differences as the majority of our respondents were female. Using the predictive mean matching method,

For peer review only - http://bmjopen.bmj.com/site/about/guidelines.xhtml

we have generated 5 complete data sets[25] and performed a pooled regression. The models were not further adjusted for non-response weights; (2) adjusted the number of care hours during lockdown analyses for the number of care hours before the pandemic; (3) explored possible deviations from the proportional odds assumption via multinomial logistic regression with the number of care hours grouped into Never (0 hours), Low (1-9 hours) and High (10 hours+).

RESULTS

Overall, 15291 participants (45% of the combined cohorts' participants) responded to the COVID-19 survey as follows: 1241 out of 1842 (NSHD), 5205 out of 8943 (NCDS), 4247 out of 10458 (BCS), 1921 out of 9380 (NS) and 2677 out of 9909 (MCS). Being female, with higher educational attainment, higher income and better self-rated health were associated with higher response rates[19].

Any participant who lacked data for at least one outcome variable was removed leaving 14891 participants that were included in the final analysis (characteristics summarized in **Table 1**). A breakdown of data missingness is presented in **Supplementary Table S1**. Overall, included participants were more likely to be female, over 50 years of age and of higher educational attainment. Older participants were more likely to have a chronic illness, receive a shielding letter, experience a cancelled appointment and require more care hours during lockdown. The chronic illnesses reported spanned a variety of medical systems (**Supplementary Table S2**). Across all cohorts, the most prevalent conditions were high blood pressure (2119 participants), recurrent back problems (1884 participants), mental health issues (1708 individuals) and asthma (1703 individuals). Individuals with multiple co-morbidities were more likely to experience cancelled surgery, medical procedures or medical appointments during lockdown and to require more care hours than those with a single chronic condition (**Supplementary Table S3**). In NS, non-White participants were less likely to be key workers (p=0.016), but we found no association between ethnicity and COVID-19 infections (p=0.296). In MCS, there was no association between ethnicity, and neither being a key worker (p=0.647), nor COVID-19 infections (p=0.979).

Cancelled surgery, medical procedures or medical appointments during lockdown

In all cohorts except NSHD, female sex was associated with higher odds (ORs range 1·20–2·29, all p<0·021) of cancelled surgery, medical procedures or medical appointments (**Table 2**). Adjusting for the presence of children less than 16 years old (**Supplementary Table S4**) and the self-rated presence of psychological distress during lockdown (**Supplementary Table S5**) attenuated the regression coefficients in most cohorts, but sex differences persisted. All the sex differences persisted after adjusting for the presence of a chronic illness, but most coefficients were attenuated (**Supplementary Table S6**). The meta-analysis revealed a pooled OR of 1·40 (95% confidence interval [CI] 1·27, 1·55) in the absence of funnel plot asymmetry (Egger test, p=0·376, **Table 3**). However, there was considerable heterogeneity between the cohorts (I^2=85·78%, p<0·0001). In each of the cohorts and in the meta-analysis, presence of a chronic illness at baseline was associated with higher odds (pooled OR 1·84 [1·65, 2·05]) of experiencing a cancelled event. The meta-analysis revealed no heterogeneity (I^2= 0·00%,

For peer review only - http://bmjopen.bmj.com/site/about/guidelines.xhtml

p=0·422) and no evidence of Funnel plot asymmetry when using the standard error as the predictor (Egger test *p*=0·092). Ethnicity and SEP were not associated with cancellations in any of the cohorts. Age was not significant in the meta-regression (**Supplementary Table S7**). A visual representation of the cancelled surgery, medical procedures or medical appointments by sex, ethnicity and the presence of chronic illness across the 5 UK cohorts is presented in **Figure 1**.

Number of care hours for self or another household member during lockdown

In older cohorts, chronic illness was more prevalent and the association with number of care hours needed was stronger (**Table 4**). In the meta-analysis, higher number of care hours was associated with ethnic minorities (OR 0·53 [0·35, 0·79], I^2=34·17%), and with the presence of chronic illness (OR 2·20 [1·72, 2·56], I^2=13·22%, **Table 5**). After adjusting for keyworker status, significant associations persisted (**Supplementary Table S8**). After adjusting for COVID-19 infection, significant associations persisted (**Supplementary Table S9**). Sex and SEP were not associated with the number of care hours needed during lockdown. There was no evidence that age contributed to the heterogeneity between cohorts from the meta-regression (**Supplementary Table S7**). Visual representation of the data is provided in **Figure 2.**

Sensitivity analysis

Associations between sex and cancelled surgery, medical procedures or other medical appointments persisted after multiple imputation (**Supplementary Table S10**). Adjustment for previous number of care hours attenuated the OR, but the associations mostly persisted (**Supplementary Table S11**). Findings were similar in the multinomial logistic regression when looking at the transition from Never (0 hours) to Low (1-9 hours), but more variability was observed at the transition from Low to High (10 hours +, **Supplementary Table S12**). When using highest educational attainment or financial difficulties before COVID-19 instead of childhood SEP, there were still no significant associations with cancellations (**Supplementary Table S13**) or the number of care hours during lockdown (**Supplementary Table S14**).

DISCUSSION

Statement of principal findings

These data from five UK national longitudinal studies, at the height of the UK national COVID-19 lockdown in May 2020, indicate worrying health inequalities in the access to health and care services–worst hit were females and those with a chronic illness or from ethnic minority groups.

Meaning of the study

Even before the COVID-19 lockdown, persons with chronic illnesses were vulnerable[26,27] and required more access to health services, as well as care from family members, friends and care service providers[28]. The pandemic triggered unprecedented changes affecting healthcare (which shifted to prioritize COVID-19 patients) and socio-economic dynamics (caused by restricted movement, changes to work patterns and remuneration and unstable housing). Our results show that persons with chronic illnesses were twice as

For peer review only - http://bmjopen.bmj.com/site/about/guidelines.xhtml

likely to have cancelled medical appointments potentially depriving them of vital medical care. They were also twice as likely to require increased number of care hours. Only around 50% of the participants had their care hours expectations met which suggests that a significant proportion were deprived of essential care. Results persisted after adjustment for shielding letter and previous care-hours illustrating their deeply rooted associations with the outcomes. Overall, participants with chronic illnesses received a double-hit with potentially long-lasting effects on their health and wellbeing. These negative effects were even more pronounced for individuals suffering with multiple co-morbidities.

We found that females were more likely to experience cancellations in planned surgery, medical procedures or other medical appointments during lockdown. This could be linked to pre-existing sex inequalities where females adopt a more caring role prioritizing other family members' needs over their own [29]. Sex inequalities during lockdown could also have widened on account of the added childcare responsibilities including home-schooling, being predominantly undertaken by women. Adjusting for the presence of children under 16 in the household attenuated the regression coefficients suggesting this was a likely contributory factor.

Ethnic minorities were twice as likely to require an increased number of care hours compared to white participants in the younger cohorts. It is likely that the unstable socio-economic landscape dominated by loss of income, unstable housing, increased psychological distress and reduced community support brought about by the lockdown restrictions adversely impacted these communities. Our care hours variable captures both home healthcare and social needs, potentially highlighting broad extra needs during lockdown. Another explanation could stem from the fact that ethnic minorities are over-represented as key workers[14]. To meet the care needs of their communities, they could have been subjected to increased working hours, unusual working environments, stricter work-based controls, and greater exposure to COVID-19, exacerbating both physical and psychological stress. However, our data suggests that ethnic minorities were under-represented as keyworkers.

Although the NHS has an extensive coverage and is free at the point of use, healthcare inequalities have been reported in the UK in the past decade [30]. An important negative finding was the absence of an association between lower socio-economic position and access to health and care services during lockdown. Speculatively, this could mean that the multiple policies implemented by the UK government to address such inequalities have paid off.

Rather surprisingly, the meta-regression showed that age was not a predictor for cancellations or accentuated care needs, suggesting an age-homogenous effect of the lockdown across the generations. We expected that older generations being more frail and likely to have received a shielding letter than younger persons, would have required more care for activities of daily-living and clinical appointments during lockdown. However, the younger generations (NS and MCS) were similarly affected in terms of medical appointments cancellations as well as the number of care hours required during

For peer review only - http://bmjopen.bmj.com/site/about/guidelines.xhtml

the pandemic. This is a potentially worrisome indication that the disruption caused by lockdown may have had far reaching effects on the health and wellbeing of young people in the UK.

Implications for clinicians and policymakers

As pandemics can be characterized by multiple waves, they can last several years[31]. Given the prospect of a third wave, it is vital that public health authorities implement national interventions to bolster health and care access. In addition, the healthcare disruptions that occurred during the first wave, are expected to lead to a surge in late-presenting conditions such as cancer[32] which will further strain the healthcare system. The challenge facing public health authorities is the need to promote access to healthcare for vulnerable groups on the one hand, whilst minimizing infection exposure on the other. Countries without a free healthcare systems where citizens rely on paid insurance such as the United States are in an even more difficult position[33].

Unanswered questions and future research

Remote healthcare known as telehealth, has been brought forward as a potential solution to the problem of health inequalities in the COVID-19 situation. However, telehealth is fraught with similar digital inequalities that will hamper the provision of equitable access[34 35]. To make telehealth egalitarian, factors contributing to digital inequalities need to be addressed. These include technical hardware disparities (lack of technological equipment, slower internet connections), digital literacy and access to technical support.

Strengths and weaknesses of the study

Strengths of the study are the implicit age homogeneity of participants enabling age-matching within each cohort as participants were exposed to similar life factors before the national lockdown. Combining five cohorts spanning multiple age groups (19, 30, 50, 62 and 74 years old) enabled a better understanding of how the COVID-19 pandemic has affected different generations. The longitudinal nature of the cohorts enabled the derivation of individualized non-response weights for each of the participants[20 21] which have been included in all analyses. This enabled us to address non-response bias rendering results more generalizable.

Limitations include data missingness due to low response rates, particularly in younger cohorts, and small sample sizes of older cohorts, particularly NSHD. As self-reported measures were used, the number of care hours needed before and during lockdown were subject to reporting biases. We binarized the ethnicity variable to enable sufficient sample sizes for comparisons, but this precluded more detailed comparisons between the diverse ethnic groups which exist in the UK. Older cohorts (NSHD, NCDS and BCS70) consist of almost only white participants, so we were unable to describe findings for older persons from minority ethnic groups that may have been most adversely affected by lockdown. Our younger cohorts (NS and MCS) include less than 20% non-White participants, which could result in specious associations. Our analysis did not take into account racism as a structural actor to explain the disparities observed and further work will be needed in future studies to address this. By considering chronic illness as a binary variable, we were unable to discriminate between minor and serious illnesses, capture multi-morbidity or

For peer review only - http://bmjopen.bmj.com/site/about/guidelines.xhtml

measure the impact spectrum generated by the cancelled appointments as well as the loss of care hours. In addition, we have not collected any data on the severity of the chronic diseases which could directly influence the need of medical appointments as well as the number of care hours required in a week. We did not capture whether participants had more than one cancelled appointment or procedure. This is especially relevant for participants from lower-socioeconomic backgrounds who are more likely to have multiple co-morbidities and may have had more than one cancelled appointment or procedure. By reducing many regression variables to a binary coding, we may have underestimated socio-economic differences which were recorded over a wider categorical spectrum. The survey question about cancelled appointments did not distinguish between face-to-face and virtual clinic consultations. We were unable to separate pandemic effects from recognized confounders such as seasonal variation in the number of care hours needed, as well as other unobserved confounders. The overall prevalence of outcomes differed between cohorts and this can affect the interpretation of ORs, potentially introducing bias between cohort comparisons. Lastly, a limitation of the restricted cubic spline meta-regression is the number of knots per variable as our study included only 5 cohorts[36].

Strength and weakness in relation to other studies

To the best of our knowledge this is the first UK study to highlight worrying health inequalities in the access to health and care services as a result of the COVID-19 pandemic. In addition, we are the first to address this issue by combining multiple cohorts spanning multiple age groups and with such a high sample size.

CONCLUSION

Individuals with a chronic illness were more likely to experience cancelled healthcare appointments and greater care needs during the UK national lockdown generated by the COVID-19 pandemic. Females experienced reduced access to healthcare, while ethnic minorities required extra care hours. Our results suggest that the pandemic might have widened pre-existing health care inequalities, further depriving already vulnerable and disadvantaged groups of the health and care services which they need. Public health measures should be rapidly implemented to better protect and meet the health and care demands of such at risk groups ahead of a COVID-19 third wave.

DATA AVAILABILITY

NSHD data is available from: https://www.nshd.mrc.ac.uk/data. Data from the remaining cohorts is available from the UK Data Archive: https://www.data-archive.ac.uk.

FUNDING

The study was funded by the Economic and Social Research Council under the Center for Longitudinal Studies, Resource Center 2015-20, grant number ES/M001660/1, and by the Medical Research Council, grant MC_UU_00019/1. G.C. is supported by British Heart Foundation (MyoFit46 Special Programme Grant SP/20/2/34841), the National Institute for Health Research Rare Diseases Translational Research Collaboration (NIHR RD-TRC) and by the NIHR UCL Hospitals Biomedical Research Center. J.C.M. is directly and indirectly supported by the UCL Hospitals NIHR BRC and Biomedical Research Unit at Barts Hospital respectively. DB is supported by the Economic and Social Research Council (grant number ES/M001660/1) and by The Academy of Medical Sciences /

For peer review only - http://bmjopen.bmj.com/site/about/guidelines.xhtml

Wellcome Trust (“Springboard Health of the Public in 2040” award: HOP001/1025). AH receives support from the British Heart Foundation, the Economic and Social Research Council (ESRC), the Horizon 2020 Framework Programme of the European Union, the National Institute on Aging, the National Institute for Health Research University College London Hospitals Biomedical Research Center, the UK Medical Research Council and works in a unit that receives support from the UK Medical Research Council. GC thanks for support the European Research Council under the European Union’s Horizon 2020 research and innovation programme (grant agreement No. 819752 DEVORHBIOSHIP – ERC-2018-COG).

ROLE OF THE FUNDING SOURCE

None of the funders was involved in the study design, the collection, the analysis, the interpretation of the data, and in the decision to submit the article for publication.

ACKNOWLEDGEMENTS

We are grateful to all the members of our studies for their contribution to this COVID-19 survey and for their ongoing participation in our studies. We thank the Survey, Data, and Administrative teams at the Center for Longitudinal Studies and Unit for Lifelong Health and Ageing, UCL, for enabling the rapid COVID-19 data collection to take place.

DISCLOSURES

The views expressed in this article are those of the authors who declare that they have no conflict of interest.

AUTHOR CONTRIBUTIONS

CT analysed the data and wrote the manuscript. CT, JCM, ADH, DB, NC, PP, G Conti and G Captur were involved in the study design and implementation. AW, ADH, DB, NC, PP and G Conti actively participated in data acquisition. G Captur contributed to the data analysis, interpretation of the results and manuscript drafting. All authors were involved in critically revising the manuscript and approved the final version. G Captur is the guarantor of this work and she attests that all listed authors meet the authorship criteria and that no others meeting the criteria have been omitted.

TRANSPARENCY STATEMENT

The authors affirm that the manuscript is an honest, accurate, and transparent account of the study being reported.

COPYRIGHT/LICENSE FOR PUBLICATION

The Corresponding Author has the right to grant on behalf of all authors and does grant on behalf of all authors, a worldwide license to the Publishers and its licensees in perpetuity, in all forms, formats and media (whether known now or created in the future), to i) publish, reproduce, distribute, display and store the Contribution, ii) translate the Contribution into other languages, create adaptations, reprints, include within collections and create summaries, extracts and/or, abstracts of the Contribution, iii) create any other derivative work(s) based on the Contribution, iv) to exploit all subsidiary rights in the Contribution, v) the inclusion of electronic links from the

For peer review only

Contribution to third party material where-ever it may be located; and, vi) licence any third party to do any or all of the above.

DISSEMINATION DECLARATION

We plan to disseminate the results to study participants.

REFERENCES

1. Bleich SN, Jarlenski MP, Bell CN, et al. Health Inequalities: Trends, Progress, and Policy. *Annual review of public health* 2012;33(1):7-40. doi: 10.1146/annurev-publhealth-031811-124658
2. Kojima G, Liljas A, Iliffe S. Frailty syndrome: implications and challenges for health care policy. *Risk management and healthcare policy* 2019;12:23-30. doi: 10.2147/rmhp.s168750
3. Mein SA. COVID-19 and Health Disparities: the Reality of "the Great Equalizer". *Journal of general internal medicine : JGIM* 2020;35(8):2439-40. doi: 10.1007/s11606-020-05880-5
4. Aspell N, apos, Sullivan M, et al. Predicting admission to long-term care and mortality among community-based, dependent older people in Ireland. *International journal of geriatric psychiatry* 2019;34(7):999-1007. doi: 10.1002/gps.5101
5. Kuh D, Pierce M, Adams J, et al. Cohort profile: updating the cohort profile for the MRC National Survey of Health and Development: a new clinic-based data collection for ageing research. *Int J Epidemiol , 40 (1) e1-e9 (2011)* 2011
6. Power C, Elliott J. Cohort profile: 1958 British birth cohort (National Child Development Study). *International journal of epidemiology* 2006;35(1):34-41. doi: 10.1093/ije/dyi183
7. Elliott J, Shepherd P. Cohort profile: 1970 British Birth Cohort (BCS70). *International Journal of Epidemiology* 2006;35(4):836-43. doi: 10.1093/ije/dyl174
8. Calderwood L, Sanchez C. Next Steps (formerly known as the Longitudinal Study of Young People in England). *Open health data* 2016;4(1):e2-e2. doi: 10.5334/ohd.16
9. Connelly R, Platt L. Cohort profile: UK Millennium Cohort Study (mcs). *International Journal of Epidemiology* 2014;43(6):1719-25. doi: 10.1093/ije/dyu001
10. Viertiö S, Kiviruusu O, Piirtola M, et al. Gender and psychological distress: contribution of work-family balance. *European journal of public health* 2019;29(Supplement_4) doi: 10.1093/eurpub/ckz187.116
11. Montazeri A, Harirchi AM, Shariati M, et al. The 12-item General Health Questionnaire (GHQ-12): translation and validation study of the Iranian version. *Health and quality of life outcomes* 2003;1(1):66-66. doi: 10.1186/1477-7525-1-66
12. Bowling A, Pikhartova J, Dodgeon B. Is mid-life social participation associated with cognitive function at age 50? Results from the British National Child Development Study (NCDS). *BMC Psychology , 4 (1) , Article 58 (2016)* 2016
13. Stolk Y, Kaplan I, Szwarc J. Clinical use of the Kessler psychological distress scales with culturally diverse groups. *International journal of methods in psychiatric research* 2014;23(2):161-83. doi: 10.1002/mpr.1426
14. CDC. Health Equity Considerations and Racial and Ethnic Minority Groups: Centers for Disease Control and Prevention; 2020 [Available from:

For peer review only

https://www.cdc.gov/coronavirus/2019-ncov/community/health-equity/race-ethnicity.html accessed 21/08/2020 2020.

15. Sze S, Pan D, Nevill CR, et al. Ethnicity and clinical outcomes in COVID-19: A systematic review and meta-analysis. *EClinicalMedicine* 2020;29-30:100630-30. doi: 10.1016/j.eclinm.2020.100630
16. Bann D, Johnson W, Li L, et al. Socioeconomic inequalities in childhood and adolescent body-mass index, weight, and height from 1953 to 2015: an analysis of four longitudinal, observational, British birth cohort studies. *The Lancet Public Health , 3 (4) (2018)* 2018
17. Elhakeem A, Cooper R, Bann D, et al. Childhood socioeconomic position and adult leisure-time physical activity: a systematic review. *The international journal of behavioral nutrition and physical activity* 2015;12(1):92. doi: 10.1186/s12966-015-0250-0
18. Brant R. Assessing Proportionality in the Proportional Odds Model for Ordinal Logistic Regression. *Biometrics* 1990;46(4):1171-78. doi: 10.2307/2532457
19. Bann D, Villadsen A, Maddock J, et al. Changes in the behavioural determinants of health during the coronavirus (COVID-19) pandemic: gender, socioeconomic and ethnic inequalities in 5 British cohort studies. medRxiv, 2020.
20. Silverwood RJ, Calderwood, L., Sakshaug, J.W., Ploubidis, G.B. A data driven approach to understanding and handling nonresponse in the Next Steps cohort. London: UCL Centre for Longitudinal Studies: CLS Working Paper 2020/5, 2020.
21. Mostafa T, Narayanan, M., Pongiglione, B., Dodgeon, B., Goodman, A., Silverwood, R.J., and Ploubidis, G.B. Improving the plausibility of the missing at random assumption in the 1958 British birth cohort: A pragmatic data driven approach. CLS Working Paper 2020/6: London: UCL Centre for Longitudinal Studies, 2020.
22. Gordon EH, Peel NM, Samanta M, et al. Sex differences in frailty: A systematic review and meta-analysis. *Experimental Gerontology* 2017;89:30-40. doi: 10.1016/j.exger.2016.12.021
23. Sterne JAC, Sutton AJ, Ioannidis JPA, et al. Recommendations for examining and interpreting funnel plot asymmetry in meta-analyses of randomised controlled trials. *BMJ* 2011;343(jul22 1):d4002-d02. doi: 10.1136/bmj.d4002
24. Orsini N, Li R, Wolk A, et al. Meta-Analysis for Linear and Nonlinear Dose-Response Relations: Examples, an Evaluation of Approximations, and Software. *American journal of epidemiology* 2012;175(1):66-73. doi: 10.1093/aje/kwr265
25. Pedersen AB, Mikkelsen EM, Cronin-Fenton D, et al. Missing data and multiple imputation in clinical epidemiological research. *Clinical epidemiology* 2017;9:157-66. doi: 10.2147/CLEP.S129785
26. Grumbach K. Chronic illness, comorbidities, and the need for medical generalism. *Annals of family medicine* 2003;1(1):4-7. doi: 10.1370/afm.47
27. Bayliss EA. Descriptions of Barriers to Self-Care by Persons with Comorbid Chronic Diseases. *Annals of family medicine* 2003;1(1):15-21. doi: 10.1370/afm.4
28. Valderas JM, Starfield B, Sibbald B, et al. Defining comorbidity: implications for understanding health and health services. *Annals of family medicine* 2009;7(4):357. doi: 10.1370/afm.983

For peer review only - http://bmjopen.bmj.com/site/about/guidelines.xhtml

29. SolÉ-AurÓ A, Crimmins EM. Who cares? A comparison of informal and formal care provision in Spain, England and the USA. *Ageing and society* 2014;34(3):495-517. doi: 10.1017/S0144686X12001134
30. Cookson R, Propper C, Asaria M, et al. Socio-Economic Inequalities in Health Care in England. *Fiscal Studies , 37 (3-4) pp 371-403 (2016)* 2016
31. He D, Dushoff J, Day T, et al. Inferring the causes of the three waves of the 1918 influenza pandemic in England and Wales. *Proceedings of the Royal Society B: Biological Sciences* 2013;280(1766):20131345. doi: 10.1098/rspb.2013.1345
32. Maringe C, Spicer J, Morris M, et al. The impact of the COVID-19 pandemic on cancer deaths due to delays in diagnosis in England, UK: a national, population-based, modelling study. *The lancet oncology* 2020;21(8):1023-34. doi: 10.1016/S1470-2045(20)30388-0
33. Khatana SAM, Groeneveld PW. Health Disparities and the Coronavirus Disease 2019 (COVID-19) Pandemic in the USA. *Journal of general internal medicine : JGIM* 2020;35(8):2431-32. doi: 10.1007/s11606-020-05916-w
34. Beaunoyer E, Dupéré S, Guitton MJ. COVID-19 and digital inequalities: Reciprocal impacts and mitigation strategies. *Computers in human behavior* 2020;111:106424. doi: 10.1016/j.chb.2020.106424
35. Crawford A, Serhal E. Digital Health Equity and COVID-19: The Innovation Curve Cannot Reinforce the Social Gradient of Health. *Journal of medical Internet research* 2020;22(6):e19361. doi: 10.2196/19361
36. Shepherd BE, Rebeiro PF. Assessing and interpreting the association between continuous covariates and outcomes in observational studies of HIV using splines. *Journal of acquired immune deficiency syndromes (1999)* 2017;74(3):e60-e63. doi: 10.1097/QAI.0000000000001221

FIGURE LEGENDS

Figure 1. Bar charts illustrating the percentages of cancelled surgery, medical appointments or other medical procedures by sex, ethnicity and the presence of chronic illness across the 5 UK longitudinal cohorts, ordered by increasing age of the cohort from left to right. Error bars representing the 95% confidence intervals are also presented.
1946 refers to National Study of Health and Development (NSHD); 1958 refers to National Child Development Study (NCDS); 1970 refers to British Cohort Study (BCS70); 1989-1990 refers to Next Steps (NS); 2000-2002 refers to Millennium Cohort Study (MCS).

Figure 2. Bar charts illustrating the percentage of participants requiring support based on the number of care hours needed during the UK COVID-19 national lockdown stratified by sex, ethnicity and the presence of chronic illness across the cohorts.

For peer review only

Table 1. Characteristics of participants by cohort.

**Participant characteristics**	**Cohort study birth year**				
	**1946**	**1958**	**1970**	**1989-1990**	**2000-2002**
Sample size					
Questionnaire respondents (*n*=15291)	1241	5205	4247	1921	2677
Included participants (*n*=14891)	1154	5119	4131	1876	2609
Age, years	74	62	50	30-31	19-20
Males, %	607 (51·88)	2432 (47·51)	1708 (41·40)	633 (34·09)	770 (29·51)
Non-white ethnicity, %	N/A	N/A	N/A	361 (19·27)	367 (14·17)
Childhood SEP I-III, %	633 (57·18)	1897 (43·60)	1727 (48·08)	1227 (69·36)	1755 (79·70)
Chronic Illness, %	842 (73.02)	3099 (61.24)	1955 (48.08)	715 (39.20)	830 (33.33)
Multi-morbidity, %	390 (33.33)	1408 (27.83)	739 (18.18)	194 (10.64)	165 (6.63)
Shielding letter, %	112 (9·61)	334 (6·57)	196 (4·77)	56 (3·00)	60 (2·30)
Presence of children <16yrs, %	0 (0·00)	87 (2·13)	1660 (41·10)	462 (25·37)	15 (0·60)
Psychological distress during lockdown, %	216 (18.77)	452 (10.25)	556 (16.07)	655 (39.15)	188 (8.29)
Key workers, %	9 (0.78)	938 (18.32)	1396 (33.79)	583 (31.08)	196 (7.51)
COVID-19 infection-self-reported or positive test, %	27 (2.31)	296 (5.78)	379 (9.18)	197 (10.50)	158 (6.06)
COVID-19 infection- positive test only, %	1 (0.09)	19 (0.37)	17 (0.41)	12 (0.64)	7 (0.27)
**Outcomes**					
Cancelled appointments, %	376 (32·58)	775 (15·17)	494 (11·97)	234 (12·47)	303 (11·61)
Care hours during lockdown					
0 hours	1073	4651	3825	1724	2552
1-4 hours	61	112	66	47	47
5-9 hours	10	41	36	10	4
10-19 hours	8	42	20	17	3
20-34 hours	5	18	16	4	2
35+ hours	13	54	35	10	1

1946 refers to National Study of Health and Development (NSHD); 1958 refers to National Child Development Study (NCDS); 1970 refers to British Cohort Study (BCS70); 1989-1990 refers to Next Steps (NS); 2000-2002 refers to Millennium Cohort Study (MCS); GCSE, general certificate of secondary education; N/A, not available; SEP, socio-economic position; yrs, years.

For peer review only

Table 2. **Association of sex, ethnicity, SEP and the presence of chronic illness with cancelled surgery, medical procedures or other medical appointments during lockdown.**

Cohort study birth year	Sex^		Ethnicity*		Socio-economic position†		Chronic Illness§	
	OR (95% CI)	*p*-value	OR (95% CI)	*p*-value	OR (95% CI)	*p*-value	OR (95% CI)	*p*-value
**1946** (*n*=1170)	0·97 (0·76, 1·25)	0·827	N/A	N/A	1·39 (0·90, 2·16)	0·138	1·74 (1·28, 2·36)	**0·0004**
**1958** (*n*=5073)	1·20 (1·03, 1·40)	**0·021**	N/A	N/A	1·05 (0·78, 1·41)	0·753	2·15 (1·76, 2·62)	**<0·0001**
**1970** (*n*=4099)	1·83 (1·47, 2·26)	**<0·0001**	N/A	N/A	1·05 (0·73, 1·51)	0·786	1·77 (1·42, 2·21)	**<0·0001**
**1989-1990** (*n*=1849)	1·70 (1·23, 2·35)	**0·001**	1·25 (0·86, 2·37)	0·255	1·45 (0·88, 2·41)	0·154	1·59 (1·18, 2·13)	**0·002**
**2000-2002** (*n*=2605)	2·29 (1·65, 3·19)	**<0·0001**	1·03 (0·73, 2·31)	0·885	1·05 (0·66, 1·67)	0·836	1·71 (1·30, 2·25)	**0·0001**

All analyses used generalized linear models with logit link. Significant *p*-values are highlighted in bold.
^ Sex was coded as 0=male and 1=female; adjustment was made for survey non-response weight and shielding letter.
* Ethnicity was coded as 0=non-White and 1=White; adjustment was made for survey non-response weight, shielding letter and sex. All participants in NSHD, NCDS and BCS were White, so ethnicity was not examined.
† Socio-economic position was coded using childhood social class from 1=managerial to 6=unskilled, but ridit scores were used; adjustment was made for survey non-response weight, shielding letter, sex and ethnicity.
§ Chronic illness was coded as 0=absent and 1=present; adjustment was made for survey non-response weight, shielding letter, sex, ethnicity and SEP.
*OR, odds ratio; CI, confidence interval. Other abbreviations as in **Table 1**.*

Table 3. **Meta-analysis for the respective association of sex and presence of chronic illness with cancelled surgery, medical procedures or other medical appointments during lockdown.**

Predictor	*n*	Study Heterogeneity			OR (95%CI)	*p*-value	Egger-test *p*-value
		I^2	Q	*p*-value			
Sex	14796	85·78%	28·12	**<0·0001**	1·40 (1·27, 1·55)	**<0·0001**	0·376
Chronic illness	12584	0·00%	3·89	0·422	1·84 (1·65, 2·05)	**<0·0001**	0·092

*Abbreviations as in **Table 1**.*

For peer review only - http://bmjopen.bmj.com/site/about/guidelines.xhtml

Table 4. Association of sex, ethnicity, socio-economic position and the presence of chronic illness with number of care hours during lockdown.

Cohort study birth year	Sex^			Ethnicity*			Socio-economic position†			Chronic Illness§		
	OR (95% CI)	*p*-value	Brant test *p*-value	OR (95% CI)	*p*-value	Brant test *p*-value	OR (95% CI)	*p*-value	Brant test *p*-value	OR (95% CI)	*p*-value	Brant test *p*-value
**1946** (n=1170)	1·17 (0·77, 1·79)	0·452	**<0·0001**	N/A	N/A	N/A	1·17 (0·56, 2·45)	0·683	0·329	2·20 (1·22, 3·99)	**0.009**	0·192
**1958** (n=4884)	1·25 (0·70, 1·61)	0·087	0·656	N/A	N/A	N/A	1·29 (0·81, 2·06)	0·282	0·877	2·17 (1·56, 3·04)	**<0·0001**	0·860
**1970** (n=3972)	0·99 (0·72, 1·36)	0·955	**0·010**	N/A	N/A	N/A	0·72 (0·39, 1·30)	0·272	0·026	2·74 (1·84, 4·08)	**<0·0001**	0·244
**1989-1990** (n=1787)	0·69 (0·44, 1·08)	0·102	**<0·0001**	0·44 (0·27, 0·72)	**0·001**	**0·005**	0·87 (0·39, 1·94)	0·727	**0·0003**	1·63 (1·01, 2·65)	**0·047**	**0·010**
**2000-2002** (n=2605)	0·88 (0·49, 1·64)	0·681	0·998	0·76 (0·38, 1·53)	0·432	0·972	2·17 (0·77, 6·25)	0·146	0·998	1·38 (0·75, 2·56)	0·301	0·961

All analyses used generalized linear models with ordinal logit link. Socio-economic was recorded using childhood social class Significant *p*-values are highlighted in bold.
^ Sex was coded as 0=male and 1=female; adjustment was made for survey non-response weight and shielding letter.
* Ethnicity was coded as 0=non-White and 1=White; adjustment was made for survey non-response weight, shielding letter and sex. All participants in NSHD, NCDS and BCS were White, so ethnicity was not examined.
† Socio-economic position was coded using childhood social class from 1=managerial to 6=unskilled, but ridit scores were used; adjustment was made for survey non-response weight, shielding letter, sex and ethnicity.
§ Chronic illness was coded as 0=absent and 1=present; adjustment was made for survey non-response weight, shielding letter, sex, ethnicity and SEP.
*Abbreviations as in **Table 1**.*

Table 5. **Meta-analysis for the respective association of sex and presence of chronic illness with number of care hours during lockdown.**

Predictor	*n*	Study Heterogeneity			OR (95%CI)	*p*-value	Egger-test *p*-value
		I^2	Q	*p*-value			
Ethnicity	4371	34·17%	0·218	0·218	0·53 (0·35, 0·79)	**0·002**	N/A^
Chronic illness	12684	13·22%	4·609	0·330	2·10 (1·72, 2·56)	**<0·0001**	0·312

^Egger test was not feasible as only 2 studies recorded ethnicity.
*Abbreviations as in **Table 1**.*

For peer review only

For peer review only - http://bmjopen.bmj.com/site/about/guidelines.xhtml

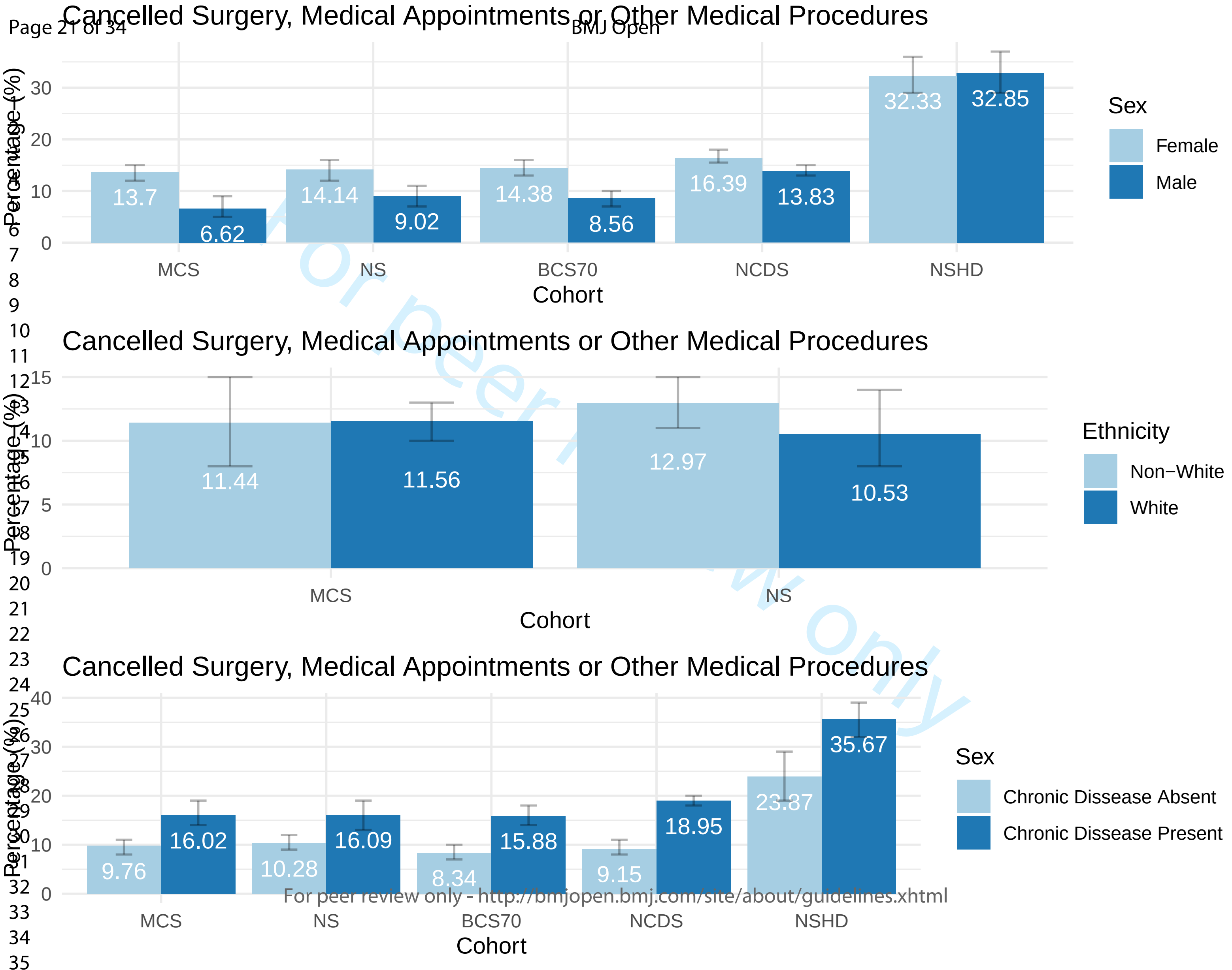

For peer review only

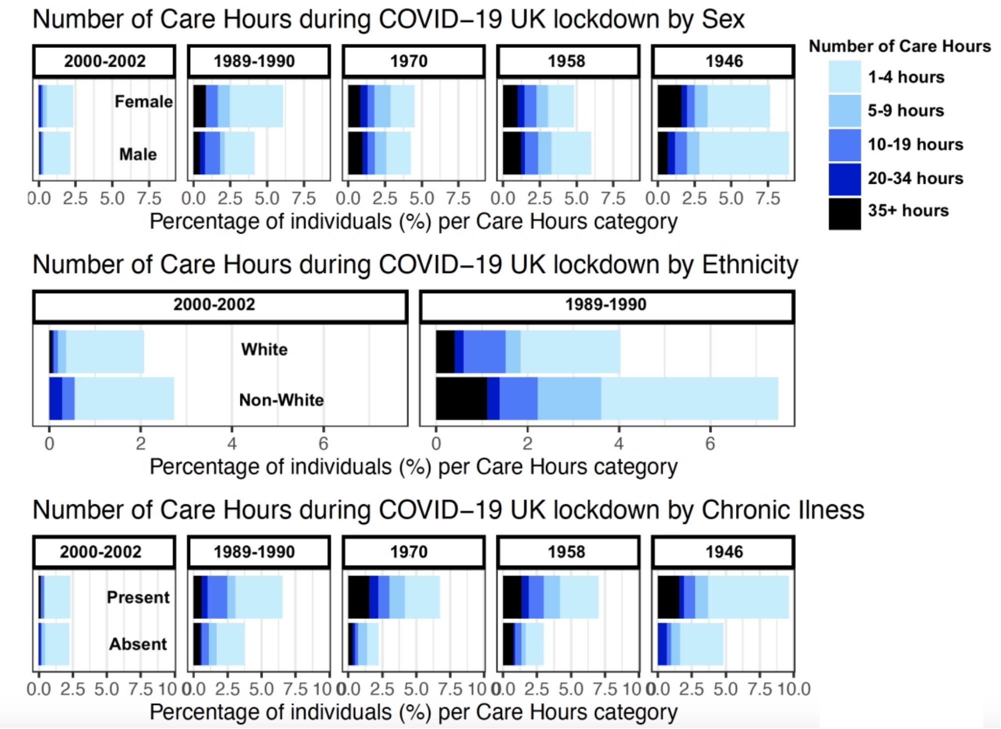

Figure 2. Bar charts illustrating the percentage of participants requiring support based on the number of care hours needed during the UK COVID-19 national lockdown stratified by sex, ethnicity and the presence of chronic illness across the cohorts.

SUPPLEMENTARY MATERIAL FOR:

Evaluating access to health and care services during lockdown by the COVID-19 survey in five UK national longitudinal studies

Constantin-Cristian Topriceanu[1,2], Andy Wong[2], James C Moon[3,4], Alun D Hughes[1,2], David Bann[5], Nish Chaturvedi[1,2], Praveetha Patalay[2,5], Gabriella Conti[6], Gabriella Captur[1,2,7]

Author Affiliations:

1. UCL Medical School, 74 Huntley St, Bloomsbury, London WC1E 6DE
2. UCL MRC Unit for Lifelong Health and Ageing, University College London, Fitzrovia, London WC1E 7HB
3. UCL Institute of Cardiovascular Science, University College London, Gower Street, London WC1E 6BT, UK
4. Cardiac MRI Unit, Barts Heart Centre, West Smithfield, London, EC1A 7BE
5. Centre for Longitudinal Studies, Department of Social Science, University College London, London WC1H 0AL, UK
6. Department of Economics, University College London, London WC1 H0AX, UK
7. The Royal Free Hospital, Centre for Inherited Heart Muscle Conditions, Cardiology Department, Pond Street, Hampstead, London NW3 2QG, UK

For peer review only

Supplementary Table S1. Data missingness per outcome.

Outcome	Cohort study birth year	Sex^	Ethnicity*	Socio-economic position†	Chronic Illness§
Cancelled surgery, medical procedures or other medical appointments during lockdown	**1946** (*n*=1170)	0	N/A	64	17
	**1958** (*n*=5073)	0	N/A	761	58
	**1970** (*n*=4099)	0	N/A	578	62
	**1989-1990** (*n*=1849)	0	2	96	49
	**2000-2002** (*n*=2605)	0	19	407	118
Number of care hours during lockdown	**1946** (n=1170)	0	N/A	64	17
	**1958** (n=4884)	0	N/A	731	53
	**1970** (n=3972)	0	N/A	554	60
	**1989-1990** (n=1787)	0	2	98	47
	**2000-2002** (n=2605)	0	19	407	118

n represents the number of individuals for which we had the corresponding outcome, the combined weight and the receipt of a shielding letter.
^ Sex missing value per outcome.
* Ethnicity missing values per outcome.
† Socio-economic position missing values per outcome.
§ Chronic illness missing values per outcome.

1946, refers to National Study of Health and Development (NSHD); 1958, refers to National Child Development Study (NCDS); 1970, refers to British Cohort Study (BCS70); 1989-1990, refers to Next Steps (NS); 2000-2002, refers to Millennium Cohort Study (MCS).

For peer review only

Supplementary Table S2. Chronic Disease breakdown.

Disease	1946	1958	1970	1989-1990	2000-2002
**Cancer**	65	147	45	4	2
**Cystic fibrosis**	0	0	0	0	1
**Asthma**	85	521	498	248	351
**COPD**	45	126	30	1	1
**Wheezy bronchitis**	13	46	21	3	3
**Diabetes**	104	404	156	21	19
**Recurrent backache/prolapsed disc/sciatica/other back problems**	217	838	587	138	104
**Hearing difficulties**	265	606	222	41	46
**Hypertension**	375	1261	443	28	12
**Heart disease**	100	236	43	2	11
**Mental health disease**	59	475	480	286	408
**Obesity**	85	542	439	154	49
**Chronic obstructive airways disease (other than COPD)**	9	23	7	1	1
**Infection**	12	53	30	14	20
**Immunodeficiency (including HIV)**	7	32	28	12	7
**Neurological disease**	24	51	51	12	12

Results are present as counts.

*COPD, chronic obstructive pulmonary disease, HIV, human immunodeficiency virus. Other abbreviations as in **Supplementary Table S1**.*

Supplementary Table S3. Comparison of participants with multiple co-morbidities vs participants with a single chronic disease in terms of the outcomes.

Cohort study birth year	Cancelled surgery, medical procedures or other medical appointments during lockdown^	Number of care hours during lockdown*
**1946**	**0·0001**	0·332
**1958**	**<0·0001**	**<0·0001**
**1970**	**<0·0001**	**<0·0001**
**1989-1990**	0·450	**0·0003**
**2000-2002**	**0·017**	0·196

P-values are presented.
^p-values were obtained from Chi squared test.
*p-values were obtained from Mann-Whitney U test.

*Abbreviations as in **Supplementary Table S1**.*

Supplementary Table S4. Associations of sex with cancelled surgery, medical procedures or other medical appointments during lockdown after adjustment for children under 16 in the household.

Cohort study birth year	Sex^	
	OR (95% CI)	*p*-value
**1946** (*n*=1154)	0·98 (0·76, 1·25)	0·839
**1958** (*n*=4940)	1·18 (1·01, 1·39)	**0·040**
**1970** (*n*=4008)	1·78 (1·45, 2·21)	**<0·0001**
**1989-1990** (*n*=1796)	1·66 (1·20, 2·34)	**0·003**
**2000-2002** (*n*=2510)	2·29 (1·66, 3·25)	**<0·0001**

Sex was coded as 0=male and 1=female.
All analyses used generalized linear models with logit link. Significant *p*-values are highlighted in bold.
^Adjustment was made for non-response weight, shielding letter and children under 16 in the household.
*CI, confidence interval; OR, odds ratio. Other abbreviations as in **Supplementary Table S1**.*

Supplementary Table S5. Associations of sex with cancelled surgery, medical procedures or other medical appointments during lockdown after adjustment for psychological distress.

Cohort study birth year	Sex^	
	OR (95% CI)	*p*-value
**1946** (*n*=1059)	1·00 (0·77, 1·30)	0·983
**1958** (*n*=4612)	1·12 (0·95, 1·32)	0·191
**1970** (*n*=3481)	1·82 (1·34, 2·30)	**<0·0001**
**1989-1990** (*n*=1697)	1·71 (1·23, 2·44)	**0·002**
**2000-2002** (*n*=2461)	2·18 (1·55, 3·11)	**<0·0001**

Sex was coded as 0=male and 1=female.
All analyses used generalized linear models with logit link. Significant *p*-values are highlighted in bold.
^Adjustment was made for non-response weight, shielding letter and psychological distress.
*Abbreviations as in **Supplementary Table S4.**.*

Supplementary Table S6. Associations of sex with cancelled surgery, medical procedures or other medical appointments during lockdown after adjustment for chronic illness.

Cohort study birth year	Sex^	
	OR (95% CI)	*p*-value
**1946** (*n*=1121)	1·01 (0·79, 1·30)	0·822
**1958** (*n*=5015)	1·24 (1·06, 1·46)	**0·007**
**1970** (*n*=4037)	1·79 (1·45, 2·21)	**<0·0001**
**1989-1990** (*n*=1800)	1·64 (1·19, 2·30)	**0·003**
**2000-2002** (*n*=2487)	2·08 (1·51, 2·93)	**<0·0001**

Sex was coded as 0=male and 1=female.
All analyses used generalized linear models with logit link. Significant *p*-values are highlighted in bold.
^Adjustment was made for non-response weight, shielding letter and chronic illness.
*Abbreviations as in **Supplementary Table S4.***

For peer review only

Supplementary Table S7. Meta-regression for the effect of age on cancelled surgery, medical procedures or other medical appointments and number of care hours needed during lockdown.

**Analysis**	**Outcome**	**n**	***p*-value^**
Cancelled appointments	Sex	14796	0·196
	Chronic Illness	12584	0·928
Number of care hours	Ethnicity*	4371	N/A
	Chronic Illness	12684	0·352

^p-values for the test of moderators for the restricted cubic spline model are provided.
*Meta-regression was not feasible as only 2 studies recorded ethnicity.
N/A, not available.

Supplementary Table S8. Associations of ethnicity with the number of care hours during lockdown after adjustment for keyworker status.

**Cohort study birth year**	**Ethnicity^**	
	**OR (95% CI)**	***p*-value**
**1989-1990** (*n*=1785)	0·45 (0·28, 0·75)	**0·002**
**2000-2002** (*n*=2586)	0·76 (0·40, 1·63)	0·453

Ethnicity was coded as 0=non-White and 1=White.
All analyses used generalized linear models with logit link. Significant *p*-values are highlighted in bold.
^Adjustment was made for non-response weight, shielding letter, sex and keyworker status.
Abbreviations as in ***Supplementary Table S4.***

Supplementary Table S9. Associations of ethnicity with the number of care hours during lockdown after adjustment for COVID-19 infection.

**Cohort study birth year**	**Ethnicity^**	
	**OR (95% CI)**	***p*-value**
**1989-1990** (*n*=1785)	0·45 (0·28, 0·74)	**0·001**
**2000-2002** (*n*=2586)	0·76 (0·39, 1·60)	0·429

Ethnicity was coded as 0=non-White and 1=White.
All analyses used generalized linear models with logit link. Significant *p*-values are highlighted in bold.
^Adjustment was made for non-response weight, shielding letter, sex and keyworker status.
Abbreviations as in ***Supplementary Table S4.***

For peer review only

Supplementary Table S10. Associations of sex and with cancelled surgery, medical procedures or other medical appointments during lockdown after multiple imputation.

Cohort study birth year	Sex^	
	**OR (95% CI)**	***p*-value**
**1958**	1·19 (1·08, 1·30)	**<0·0001**
**1970**	1·83 (1·61, 2·07)	**<0·0001**
**1989-1990**	1·50 (1·23, 1·83)	**0·002**
**2000-2002**	1·75 (1·32, 2·33)	**0·003**

Sex was coded as 0=male and 1=female.
All analyses used generalized linear models with logit link. Significant *p*-values are highlighted in bold.
^ Adjustment was made for shielding letter.
Abbreviations as in ***Supplementary Table S4.***

For peer review only

Supplementary Table S11. Association of sex, ethnicity, socio-economic position and the presence of chronic illness with number of care hours during lockdown after further adjustment for hours of care before lockdown.

Cohort study birth year	Sex^			Ethnicity*			Socio-economic position†			Chronic Illness§		
	OR (95% CI)	*p*-value	Brant test *p*-value	OR (95% CI)	*p*-value	Brant test *p*-value	OR (95% CI)	*p*-value	Brant test *p*-value	OR (95% CI)	*p*-value	Brant test *p*-value
**1946** (*n*=1170)	1·25 (0·76, 2·07)	0·377	**<0·0001**	N/A	N/A	N/A	0·85 (0·35, 2·06)	0·718	0·148	1·93 (1·02, 4·00)	**0·048**	0·260
**1958** (*n*=4860)	1·42 (1·02, 1·99)	**0·038**	**0·004**	N/A	N/A	N/A	1·22 (0·66, 2·26)	0·523	**0·046**	1·59 (1·05, 2·45)	**0·031**	**0·015**
**1970** (*n*=3960)	0·91 (0·61, 1·37)	0·654	**<0·0001**	N/A	N/A	N/A	1·37 (0·67, 2·84)	0·389	**<0·0001**	1·75 (1·10, 2·81)	**0·020**	**<0·0001**
**1989-1990** (*n*=1782)	0·60 (0·33, 1·10)	0·097	0·153	0·55 (0·29, 1·11)	0·086	**<0·0001**	1·34 (0·48, 3·83)	0·457	**<0·0001**	1·61 (0·87, 2·98)	0·128	**<0·0001**
**2000-2002** (*n*=2478)	0·95 (0·51, 1·81)	0·868	0·967	0·70 (0·32, 1·76)	0·404	1·00	2·18 (0·76, 6·42)	0·148	0·998	1·21 (0·62, 2·28)	0·562	0·938

Sex was coded as 0=male and 1=female and ethnicity as 0=non-White and 1=White· Socio-economic was coded using childhood social class from 1=managerial to 6=unskilled, but ridit scores were used. Chronic illness was coded as 0=absent and 1=present.

All analyses used generalized linear models with ordinal logit link. Significant *p*-values are highlighted in bold.

^ Adjustment was made for survey non-response weight, previous care hours and shielding letter.

* Adjustment was made for survey non-response weight, previous care hours, shielding letter and sex.

† Adjustment was made for survey non-response weight, previous care hours, shielding letter, sex and ethnicity.

§ Adjustment was made for survey non-response weight, previous care hours shielding letter, sex, ethnicity and SEP.

N/A, not available. Other abbreviations as in ***Supplementary Table S4.***

For peer review only

Supplementary Table S12. Association of sex, ethnicity, socio-economic position and the presence of chronic illness with number of care hours during lockdown using multinomial logistic regression.

	Sex^			Ethnicity*			Socio-economic position[†]			Chronic Illness[§]		
**Cohort study birth year**	**Never[ø]**	**Low RRR [⸝] (95% CI)**	**High RRR [˃] (95% CI)**	**Never**	**Low RRR (95% CI)**	**High RRR (95% CI)**	**Never**	**Low RRR (95% CI)**	**High RRR (95% CI)**	**Never**	**Low RRR (95% CI)**	**High RRR (95% CI)**
**1946** (*n*=1170)	ref	1·30 (0·79, 2·17)	0·82 (0·38, 1·81)	N/A	N/A	N/A	ref	0·93 (0·39, 2·19)	2·18 (0·55, 8·70)	ref	1·90 (0·97, 3·72)	3·15 (0·93, 10·28)
**1958** (*n*=4884)	ref	1·34 (0·67, 1·50)	1·01 (0·94, 1·92)	N/A	N/A	N/A	ref	1·34 (0·94, 1·91)	1·24 (0·62, 2·48)	ref	**2·27 (1·46, 3·53)**	**2·08 (1·27, 3·40)**
**1970** (*n*=3972)	ref	1·02 (0·66, 1·60)	1·27 (0·72, 2·23)	N/A	N/A	N/A	ref	0·74 (0·35, 1·57)	0·69 (0·27, 1·77)	ref	**2·07 (1·28, 3·36)**	**4·39 (2·18, 8·84)**
**1989-1990** (*n*=1787)	ref	0·49 (0·27, 0·89)	1·11 (0·49, 2·51)	ref	**0·73 (0·20, 0·70)**	0·37 (0·27, 1·99)	ref	0·64 (0·24, 1·76)	1·41 (0·36, 5·43)	ref	1·53 (0·84, 2·78)	1·83 (0·83, 4·05)
**2000-2002** (*n*=2605)	ref	0·50 (0·44, 1·84)	0·90 (0·01, 3·53)	ref	0·92 (0·01, 1·6)	0·19 (0·36, 2·36)	ref	1·98 (0·19, 11·16)	4·62 (0·66, 5·97)	ref	1·08 (0·55, 2·12)	**9·24 (1·03, 83·25)**

Sex was coded as 0=male and 1=female and ethnicity as 0=non-White and 1=White· Socio-economic was coded using childhood social class from 1=managerial to 6=unskilled, but ridit scores were used. Chronic illness was coded as 0=absent and 1=present.

All analyses used multinomial logistic regression.

Statistically significant findings (95% CI exclude the null value) are highlighted in bold.

^ Adjustment was made for survey non-response weight and shielding letter.

* Adjustment was made for survey non-response weight, shielding letter and sex.

[†] Adjustment was made for survey non-response weight, shielding letter, sex and ethnicity.

[§] Adjustment was made for survey non-response weight shielding letter, sex, ethnicity and SEP.

[ø] Defined as 0 hours of care needed post-COVID-19.

[⸝] Defined as 1-9 hours of care needed post-COVID-19.

[˃] Defined as 10+ hours of care needed post-COVID-19.

ref, reference; RRR, relative risk ratio. Other abbreviations as in ***Supplementary Table S4.***

For peer review only

Supplementary Table S13. Association of socio-economic position surrogates with cancelled surgery, medical procedures or other medical appointments during lockdown.

Cohort study birth year	SEP as highest educational attainment ridit score^			SEP as pre-COVID financial difficulties ridit score^		
	n	**OR (95% CI)**	***p*-value**	***n***	**OR (95% CI)**	***p*-value**
**1946**	1089	1·43 (0·92, 2·21)	0·111	1145	1·28 (0·84, 1·97)	0·246
**1958**	4972	1·09 (0·83, 1·42)	0·559	4878	1·11 (0·85, 1·46)	0·454
**1970**	3850	1·12 (0·80, 1·58)	0·501	3978	1·10 (0·79, 1·54)	0·576
**1989-1990**	1722	1·41 (0·84, 2·37)	0·190	1768	1·49 (0·91, 2·44)	0·119
**2000-2002**	2444	0·84 (0·54, 1·31)	0·477	2413	0·90 (0·58, 1·40)	0·654

All analyses used generalized linear models with logit link.
^ Adjustment was made for survey non-response weight, shielding letter, sex and ethnicity.
Abbreviations as in ***Supplementary Table S4.***

For peer review only

Supplementary Table S14. Association of socio-economic position surrogates with the number of care hours during lockdown.

Cohort study birth year	SEP as highest educational attainment ridit score^			SEP as pre-COVID financial difficulties ridit score^		
	n	**OR (95% CI)**	***p*-value**	***n***	**OR (95% CI)**	***p*-value**
**1946**	1170	1·17 (0·77, 1·79)	0·672	1170	1·18 (0·58, 2·40)	0.657
**1958**	4789	1·12 (0·72, 1·75)	0·609	4836	1·20 (0·77, 1·87)	0·407
**1970**	3747	0·68 (0·39, 1·20)	0·182	3954	0·81 (0·47, 1·39)	0·345
**1989-1990**	1666	0·99 (0·43, 2·25)	0·979	1768	0·92 (0·43, 2·00)	0·841
**2000-2002**	2444	1·53 (0·57, 4·14)	0·408	2420	1·86 (0·73, 4·79)	0·196

All analyses used generalized linear models with ordinal logit link·
^ Adjustment was made for survey non-response weight, shielding letter, sex and ethnicity.
*Abbreviations as in **Supplementary Table S4.***

For peer review only

STROBE CHECKLIST FOR:

Inequality of access to health and care services during lockdown–Findings from the COVID-19 survey in five UK national longitudinal studies

Constantin-Cristian Topriceanu[1,2], Andy Wong[2], James C Moon[3,4], Alun D Hughes[1,2], David Bann[5], Nish Chaturvedi[1,2], Praveetha Patalay[2,5], Gabriella Conti[6], Gabriella Captur[1,2,7]

Author Affiliations:

1. UCL Medical School, 74 Huntley St, Bloomsbury, London WC1E 6DE
2. UCL MRC Unit for Lifelong Health and Ageing, University College London, Fitzrovia, London WC1E 7HB
3. UCL Institute of Cardiovascular Science, University College London, Gower Street, London WC1E 6BT, UK
4. Cardiac MRI Unit, Barts Heart Centre, West Smithfield, London, EC1A 7BE
5. Centre for Longitudinal Studies, Department of Social Science, University College London, London WC1H 0AL, UK
6. Department of Economics, University College London, London WC1 H0AX, UK
7. The Royal Free Hospital, Centre for Inherited Heart Muscle Conditions, Cardiology Department, Pond Street, Hampstead, London NW3 2QG, UK

For peer review only - http://bmjopen.bmj.com/site/about/guidelines.xhtml

STROBE Statement—Checklist of items that should be included in reports of ***cohort studies***

	Item No	Recommendation	Page
**Title and abstract**	1	(*a*) Indicate the study's design with a commonly used term in the title or the abstract	**1**
		(*b*) Provide in the abstract an informative and balanced summary of what was done and what was found	**2**
**Introduction**			
Background/rationale	2	Explain the scientific background and rationale for the investigation being reported	**4**
Objectives	3	State specific objectives, including any prespecified hypotheses	**4**
**Methods**			
Study design	4	Present key elements of study design early in the paper	**4**
Setting	5	Describe the setting, locations, and relevant dates, including periods of recruitment, exposure, follow-up, and data collection	**4**
Participants	6	(*a*) Give the eligibility criteria, and the sources and methods of selection of participants. Describe methods of follow-up	**5**
		(*b*) For matched studies, give matching criteria and number of exposed and unexposed	**Yes**
Variables	7	Clearly define all outcomes, exposures, predictors, potential confounders, and effect modifiers. Give diagnostic criteria, if applicable	**5**
Data sources/ measurement	8*	For each variable of interest, give sources of data and details of methods of assessment (measurement). Describe comparability of assessment methods if there is more than one group	**5**
Bias	9	Describe any efforts to address potential sources of bias	**4,5,6**
Study size	10	Explain how the study size was arrived at	**5,6**
Quantitative variables	11	Explain how quantitative variables were handled in the analyses. If applicable, describe which groupings were chosen and why	**5,6**
Statistical methods	12	(*a*) Describe all statistical methods, including those used to control for confounding	**5,6**
		(*b*) Describe any methods used to examine subgroups and interactions	**5,6**
		(*c*) Explain how missing data were addressed	**5,6**
		(*d*) If applicable, explain how loss to follow-up was addressed	**5,6**
		(*e*) Describe any sensitivity analyses	**5,6**
**Results**			
Participants	13*	(a) Report numbers of individuals at each stage of study—eg numbers potentially eligible, examined for eligibility, confirmed eligible, included in the study, completing follow-up, and analysed	**7**
		(b) Give reasons for non-participation at each stage	**7**
		(c) Consider use of a flow diagram	**N/A**
Descriptive data	14*	(a) Give characteristics of study participants (eg demographic, clinical, social) and information on exposures and potential confounders	**7**
		(b) Indicate number of participants with missing data for each variable of interest	**7**
		(c) Summarise follow-up time (eg, average and total amount)	**4**
Outcome data	15*	Report numbers of outcome events or summary measures over time	**7**

For peer review only

Main results	16	(*a*) Give unadjusted estimates and, if applicable, confounder-adjusted estimates and their precision (eg, 95% confidence interval). Make clear which confounders were adjusted for and why they were included	**7**
		(*b*) Report category boundaries when continuous variables were categorized	**7**
		(*c*) If relevant, consider translating estimates of relative risk into absolute risk for a meaningful time period	**N/A**
Other analyses	17	Report other analyses done—eg analyses of subgroups and interactions, and sensitivity analyses	**8**
**Discussion**			
Key results	18	Summarise key results with reference to study objectives	**9**
Limitations	19	Discuss limitations of the study, taking into account sources of potential bias or imprecision. Discuss both direction and magnitude of any potential bias	**10**
Interpretation	20	Give a cautious overall interpretation of results considering objectives, limitations, multiplicity of analyses, results from similar studies, and other relevant evidence	**9**
Generalisability	21	Discuss the generalisability (external validity) of the study results	**9.10**
**Other information**			
Funding	22	Give the source of funding and the role of the funders for the present study and, if applicable, for the original study on which the present article is based	**10,11**

*Give information separately for exposed and unexposed groups.

For peer review only
